# Altered Immunity in Crowded Locust Reduced Fungal (*Metarhizium anisopliae*) Pathogenesis

**DOI:** 10.1371/journal.ppat.1003102

**Published:** 2013-01-10

**Authors:** Yundan Wang, Pengcheng Yang, Feng Cui, Le Kang

**Affiliations:** State Key Laboratory of Integrated Management of Pest Insects and Rodents, Institute of Zoology, Chinese Academy of Sciences, Beijing, China; Stanford University, United States of America

## Abstract

The stress of living conditions, similar to infections, alters animal immunity. High population density is empirically considered to induce prophylactic immunity to reduce the infection risk, which was challenged by a model of low connectivity between infectious and susceptible individuals in crowded animals. The migratory locust, which exhibits polyphenism through gregarious and solitary phases in response to population density and displays different resistance to fungal biopesticide (*Metarhizium anisopliae*), was used to observe the prophylactic immunity of crowded animals. We applied an RNA-sequencing assay to investigate differential expression in fat body samples of gregarious and solitary locusts before and after infection. Solitary locusts devoted at least twice the number of genes for combating *M. anisopliae* infection than gregarious locusts. The transcription of immune molecules such as pattern recognition proteins, protease inhibitors, and anti-oxidation proteins, was increased in prophylactic immunity of gregarious locusts. The differentially expressed transcripts reducing gregarious locust susceptibility to *M. anisopliae* were confirmed at the transcriptional and translational level. Further investigation revealed that locust GNBP3 was susceptible to proteolysis while GNBP1, induced by *M. anisopliae* infection, resisted proteolysis. Silencing of *gnbp3* by RNAi significantly shortened the life span of gregarious locusts but not solitary locusts. By contrast, *gnbp1* silencing did not affect the life span of both gregarious and solitary locusts after *M. anisopliae* infection. Thus, the GNBP3-dependent immune responses were involved in the phenotypic resistance of gregarious locusts to fungal infection, but were redundant in solitary locusts. Our results indicated that gregarious locusts prophylactically activated upstream modulators of immune cascades rather than downstream effectors, preferring to quarantine rather than eliminate pathogens to conserve energy meanwhile increasing the “distance” of infectious and target individuals. Our study has obvious implications for bio-pesticides management of crowded pests, and for understanding disease epidemics and adaptiveness of pathogens.

## Author Summary

The wide application of fungal biopesticides for insect management has led to concerns over the development of biopesticide resistance. The migratory locust, a globally notorious agricultural pest, has density-dependent phase changes between solitary and gregarious states. The gregarious locusts displayed longer life spans than solitary locusts after biopesticide *Metarhizium anisopliae* infection. We analyzed prophylactic immunity of the locusts in phase change adaptation by transcriptome analysis. Gregarious locusts optimized immunity by investing more in molecules of upstream immune cascades including pattern recognition proteins, anti-oxidation proteins, protease inhibitors and serine protease. High levels of pattern recognition proteins guided deposition of immune products onto pathogens reducing growth, proliferation and transmission. This prophylactic immunity of gregarious locusts emphasized on quarenteening *M. anisopliae* pathogens in early infection, which decreased individuals' infection risk in a population and avoids disease epidemics. Pest outbreaks mostly occur in high population densities, thereby, diminishing entomopathogen biopesticide efficiency. Our results provide an insight to an organism's “enhanced” immunity induced by population densities and inspires new paradigms to understand biopesticide tolerance and disease epidemics in the future.

## Introduction

Similar to pathogenic infection, the stress of living conditions alters animal immunity. Changes in population density usually induce polyphenic transition and variations in the immune response of animals [1]. The locust, globally notorious agricultural pests that have been controlled with fungal biopesticides for decades, exhibits phenotypic changes during solitary or gregarious phases in response to low or high population densities, respectively [2]. Differences in morphology, behavior and physiology have been observed between the two locust phases. Gregarious locusts (*Schistocerca gregaria*) have longer life spans than solitary locusts in response to treatment with a lethal fungal biopesticide (*Metarhizium anisopliae var. acridum*) [3]. The altered susceptibility of gregarious locusts through changes in population density potentially enhance the risk of fungal biopesticide resistance and provide an ideal model system for investigating the environmental factors that modulate the phenotypic immunity of locusts.

High population densities increase the risk of infection by individuals through contact and injury from cannibalism, which require relatively higher investments into host immunity [4], [5], [6]. However, several gregarious insects display low total hemocyte counts and decreased phenoloxidase (PO) activity, previously postulated to have arisen from the increasing the “distance” between the susceptible individuals and infected individuals, due to the gap filled by more healthy individuals after crowding, resulting in a relatively lower risk of infection [7]. This assumption from Watve's model depends on the increasing Euclidean or behavioral distance between the susceptible individuals and infected individuals [8], [9], [10]. That is, any physiological or behavioral characteristics of host (such as rapid wound healing after injury or cannibalism, allogrooming after pathogen attachment to body surfaces and other hygienic activities) beneficial to inhibiting output of pathogens particles from infectious individuals and increasing the “distance” between hosts ensures the health of crowded individuals. Previous observations of altered immunity in high-density populations were largely incomprehensive because the prophylactic investment in immunity could be emphasized either on behavioral defenses [11] or physiological defense, displaying the defense strategies from pathogens elimination to the control of pathogens proliferation, spread and damages [12], [13], [14], [15]. Therefore, it is essential to find indicators for the evaluation of density dependent prophylaxis either in physiological or behavioral defenses.

Unlike behavioral defenses, physiological defenses are largely depend on distinctive, efficient, and dedicated immune responses. When fungi germinate on insect integument and penetrate into their hemocoel, fungal molecular patterns (mostly β-1,3-glucans) and virulent factors (PR1) from the growing hyphae are detected by insect immune surveillance molecules such as glucan recognition proteins (GNBP/GRP) and persephone (PSH). In response to the fungal invasion, the insect immune system initiates series of defenses such as humoral melanization, fungal β-1,3-glucan degradation, assembly of attack complex and production of intracellular antimicrobial peptides (AMPs) [16], [17]. The relatively simple immune system of invertebrates allows for the comprehensive observation of resource allocation in immune cascades and for further understanding of the host defense strategy in response to changes in population density.

Previous studies have attempted to understand desert locust phenotypic immunity by comparing the differences in behavioral fever, hemocyte counts, bacterial lysis and melanization [3]. However, the migratory locust modulated behavioral phase changes through different molecules comparing with desert locusts in response to population density changes [18], [19], [20], [21]. Moreover, the detailed molecular mechanisms underlying the phenotypic response of either in the desert or the migratory locust to *M. anisopliae* infection are rarely observed [22], [23]. Our previous work found that locust innate immune genes of the Toll and IMD pathway existed in the expressed sequence tag (EST) library. In addition, the software for *de novo* short reads assembly from RNA-sequencing (RNA-seq) without genome references were successfully developed [24], [25]. These fundamental studies provide a molecular basis for understanding the phenotypic resistance of locusts to *M. anisopliae*. With the knowledge that the fat body is the major insect organ for immune responses and energy metabolism [26], [27], RNA-sequencing transcriptome analysis was used to investigate the mRNA expression profiles in fat bodies of basal and *M. anisopliae*-infected two phases of the migratory locusts (*Locusta migratoria*), in order to determine whether increased population density induces phenotypic immunecompetence in locust. The differentially expressed transcripts involved in phase changes and resistance to *M. anisopliae* were determined via *de novo* assembly in combination with expression abundance calculations using Trinity software [25]. The full-length cDNAs of DETs were confirmed and their expression patterns were determined at the transcriptional level using quantitative real-time polymerase chain reaction (PCR). The target genes were knocked down via the dsRNA method to analyze their effects on phenotypic resistance to fungal infection and in modulating immune cascades. The present study revealed important information for understanding how resistance against *M. anisopliae* is modulated at the molecular level by density-dependent prophylaxis.

## Results

### Gregarious locusts have phenotypically diminished *M. anisopliae* susceptibility

**Figure 1:**
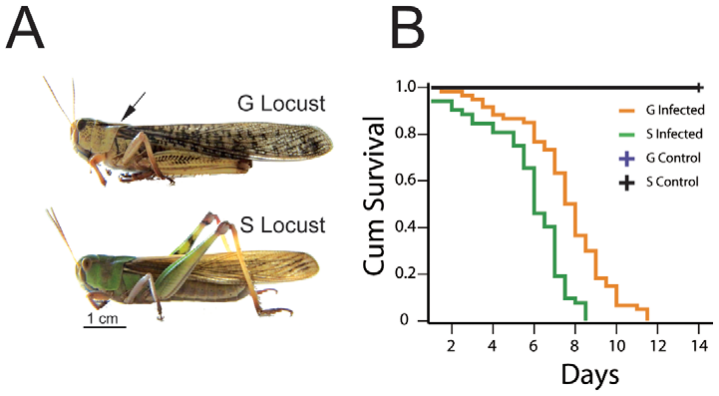
Locust phenotypic life span after lethal fungal infection (*M. anisopliae*). (A) Adult gregarious and solitary locusts were topically infected with fungi (*M. anisopliae*) under the protonum to prevent septic injury. The arrow indicates the inoculation site. Scale bar: 1 cm. (B) Gregarious (G) and solitary (S) locusts were randomly selected (female G:20, S:24 and male G:40, S:27) for fungal infection assay. The life span of the locusts was calculated by Kaplan-Meier methods, and Cox proportional hazards model analysis was used for assessing variables affecting locusts survival. The curves of control treatments (blue and black lines) largely overlapped and displayed as a single black line. (Three replicates for each locust phase, χ^2^ = 25.959, *P<*0.001).

Fungal inoculation (*M. anisopliae*, IMP003) was performed under the protonum to avoid septic injury (**Figure 1A**). Analyzing the variables that affect locust survival, indicated that sex (χ^2^ = 0.598, *P = *0.439) and body weight (χ^2^ = 1.69, *P* = 0.193) had negligible effects on locust survival, whereas the phase variable (solitary to gregarious phase) increased 2.6 times hazard ratio (95%CI 0.259–0.590, χ^2^ = 22.581, *P*<0.001) of locust survival (**Table S1**). Adult solitary and gregarious locusts exhibited significantly different life spans (χ^2^ = 25.959, *P*<0.001) under Kaplan-Meier analysis (**Figure 1B**). The mean life span of the gregarious locusts was 1d to 2d longer than that of solitary locusts (gregarious locusts, 7.5±0.2; solitary locusts, 6.0±0.3). The lifespan of the migratory locust was correlated with the phase change rather than gender or body weight.

### RNA-sequencing analysis of differentially expressed transcripts before and after *M. anisopliae* infection in solitary and gregarious locusts

The *M. anisopliae* infection bioassay confirmed that gregarious locusts have longer life spans than the solitary locusts. To investigate candidate genes involved in locust phenotypic immunity, we used RNA-seq transcriptome analysis to observe the responses to fungal infections of solitary and gregarious locusts. After sequencing >140 million pair-end reads of fat body samples before and after infected, Trinity software was used for the *de novo* assembly of transcripts and fat body samples from infected gregarious locusts was used as reference transcripts (N50 = 967 bp, longest transcripts = 12,677 bp) to align reads for calculating DETs (**Table S2**).

**Figure 2:**
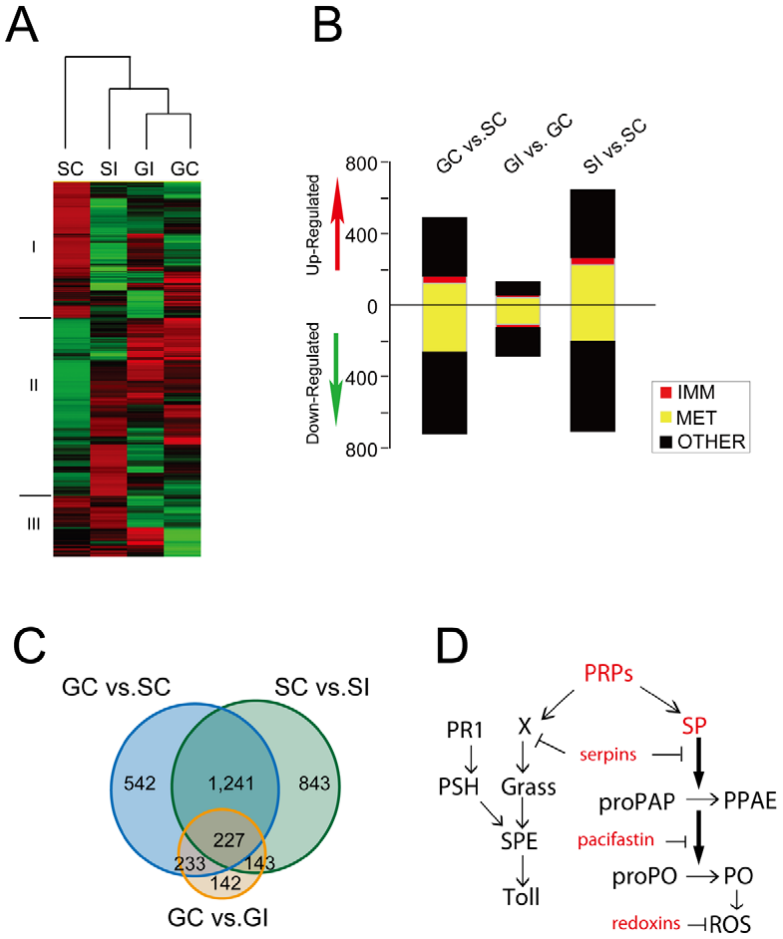
Transcriptome analysis of the fatbody of gregarious and solitary locusts before and after infection. The abundance and differential expression of transcripts were detected by software packages of Trinity and DEGseq respectively after assembling reference transcripts from raw reads from illumine Truseq experiments. (A) Hierarchical cluster analysis of fatbody transcripts that were significantly regulated (*P*<0.001, q-value<0.05) in at least two samples of four experimental conditions, and two phase locusts displayed distinct response to fungal infection. Heatmap was calculated by implement package of Trinity software. (B) Differential expressed transcripts (*P*<0.001, q-value<0.05) were classed by function (blast2go 1.0E-6). GC: fatbody sample of control gregarious locusts; SC: fatbody sample of control solitary locusts; GI: fatbody sample of gregarious locust infected by *M. anisopliae*; SI: fatbody sample of solitary locusts infected by *M. anisopliae*. IMM: immune defenses; MET: metabolism functions; OTHER: other biological process including unidentified. (C) Venn diagram representing unique and shared transcriptome regulation of prophylactic immunity and responsiveness of the two phases of locust to *M. anisopliae* (D) Prophylactically presented immune molecules of gregarious locust were showed as red in schematic immune pathways of resistance to fungal infection. PRPs: pattern recognition proteins; SP: serine protease; proPAP: pro-phenoloxidase activating proteinase; PPAE: proPO activating enzyme; PO: phenoloxidase; ROS: reactive oxygen species; Serpins: serine protease inhibitors; Grass: Gram-positive Specific Serine protease; SPE: Spätzle-processing enzyme; PSH: Persephone; PR1: fungal virulence protease PR1.

After DETs detected were by DEGseq software, hierarchical clustering analysis of total DETs (3,418 DETs, q-value<0.05, P<0.001) indicated that the two phases of the locust resisted the *M. anisopliae* infection with different strategies (**Figure 2A**). Before *M. anisopliae* infection, gregarious locusts highly expressed immune molecules including pattern recognition proteins (PRPs), serine proteases, serine protease inhibitors (serpins and pacifastin), reactive oxygen species (ROS) inhibitors (peroxiredoxin) and cellular surface molecules (CD-like proteins) (**Data S1**). In response to *M. anisopliae* infection, the solitary locusts increased the expression of genes related to behavior, proteolysis, protease inhibition, oxidation/reduction and signal transduction, but the gregarious locusts already had increased the expression of these genes (**Figure 2B, 2C** **and Data S1**). *M. anisopliae* stimulated expression of at least twice the numbers of genes in the solitary locust than in gregarious locust as determined by two independent software analysis (**Figure S1 and S2, Table S3 and S4**). In dual detection pathway of fungal infection [28], we found that prophylaxis immunity of gregarious locusts focused on upstream modulators of the pathway triggered by GNBPs (**Figure 2D**). Interestingly, expression of cellular surface molecules (CD-like proteins) was increased after *M. anisopliae* infection. The *drosomycin*-like specific anti-fungal peptide transcripts were not observed in the locusts before and after fungal infection. This could be due to our cutoff limit of 180 bp in assembling reference transcripts.

Although various insect immune molecules were previously investigated in altered immunity [29], [30], [31], prophylactic expression of PRPs (GNBPs and PGRPs) rather than downstream products such as antimicrobial peptides lead us to understand the underlying mechanisms of GNBPs in modulating phenotypic fungal-resistance at top of immune cascades. We identified three *gnbp* homologous genes from *de novo* assembled transcripts and confirmed their full-length cDNA sequences by using rapid amplification of cDNA ends (RACE) method. *De novo* assembly of Trinity software successfully discerned the homologous transcripts that were consistent with the RACE results. The subsequent phylogenetic analysis of the deduced amino acid sequences showed that locust GNBP1 and GNBP3 are recognition proteins whereas GNBP2 is a putative glucanase protein (**Figure S3**).

### Pattern recognition proteins were prophylactically expressed in response to phase change

**Figure 3:**
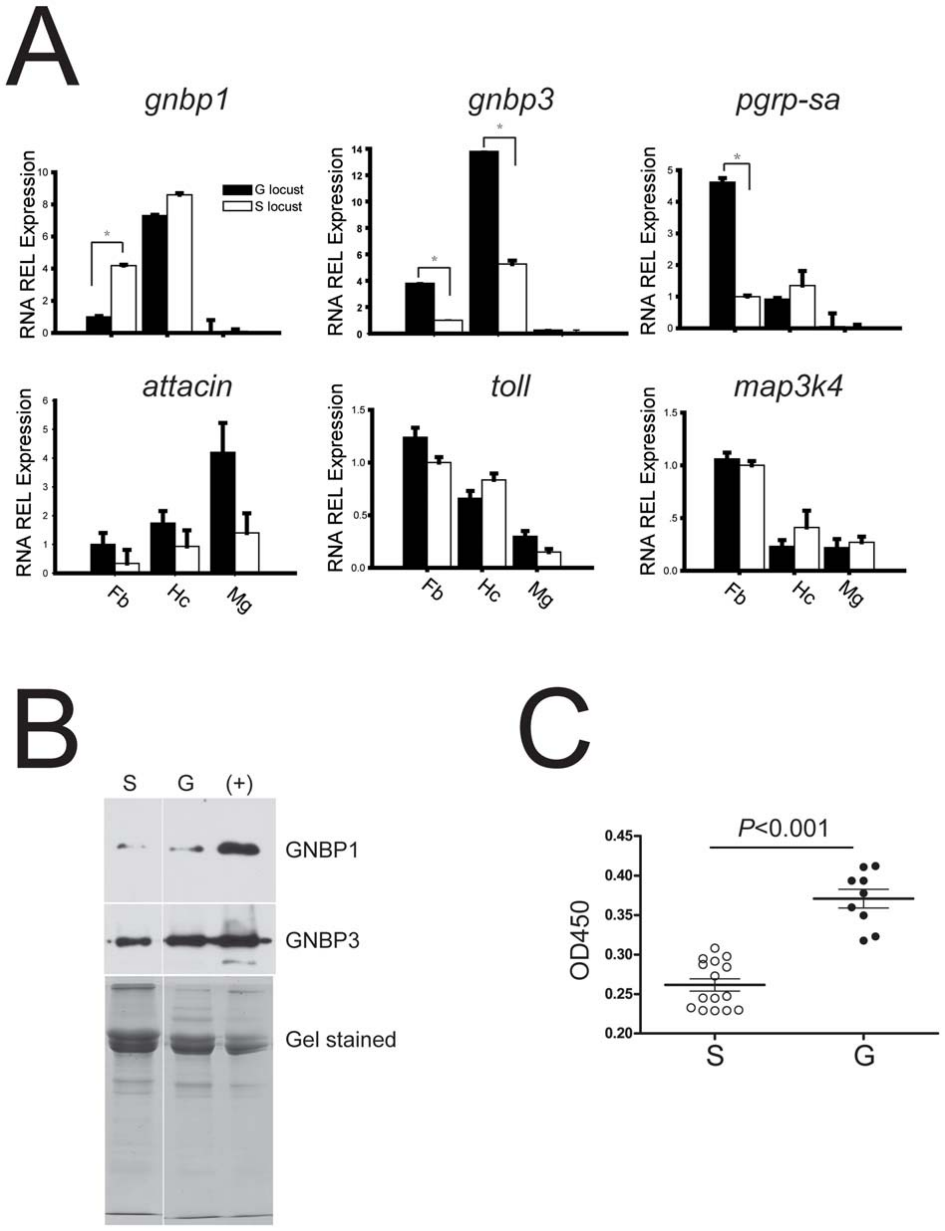
Determination of prophylactically expressed immune genes between two phase locust. (A) Differential expressed transcripts between pre-infected solitary and gregarious locusts in main immune tissues (Fb: fat bodies; Hc: hemocytes; Mg: midgut) were confirmed by quantitative real time PCR. Data was presented as mean (±SE) and analyzed by Mann-Whitney U test (B) Immunoblot assay examination of circulating GNBPs between pre-infected two phase locusts. Upper panel indicated the immunoblot results of polyantibodies against locust GNBP1 and GNBP3 respectively; Lower panel was coomassie brilliant blue stained SDS-PAGE for protein amount calibration; positive sample was fungi infected locust hemolymph after 4 days. S: solitary locust; G: gregarious locust. (C) The circulating GNBP3 in hemolymph of pre-infected two phase locusts was measured by ELISA assay. G: gregarious locust; S: solitary locust. Briefly, polyantibodies against C-terminal of GNBP3 was coated on plate to capture circulating GNBP3, and polyantibodies against N-terminal of GNBP3 were conjugated with HRP to recognize the captured GNBP3 proteins. OD450 values indicated the relative abundance of GNBP3 in hemolymph, total hemolymph proteins were used for calibration (one-way ANOVA, *F* = 12.61, *P* = 0.002).

Quantitative real-time PCR of selected genes from immune cascades was performed to confirm the consistency (*r^2^* ^= ^0.85) of differential expression with transcriptome analysis (**Table S5**). The data revealed significant differential expressions of *gnbp1* (n = 18, two tailed *P*<0.001), *gnbp3* (n = 18, two tailed *P*<0.001), and *pgrp-sa* (n = 15, two tailed *P*<0.001) between the two locust phases without *M. anisopliae* infection (**Figure 3A**). Although *attacin* production was already considered as specific for insect immune defense, the differential expression of *toll* and downstream *attacin* were not detected in fat bodies between the two locust phases. The toll receptor, a cellular membrane protein that triggers intracellular responses to fungal infection, showed no significantly different expressions between the two locust phases while *cactus* for inhibiting downstream immune effectors production was highly presented (n = 12, two tailed *P*<0.001) in fat body of gregarious locusts (**Table S5**). In addition, there was no significant difference in the expression of *map3k4* in response to temporary stress between the two locust phases. The phenotypic expression of pacifastin-like proteins was consistent with a previous observation in the two locust phases (**Data S1**) [32], [33]. Although solitary locusts showed enhanced mRNA expression of GNBP1 in fat bodies compared to gregarious locusts (**Figure 3A**), further immunoblot analysis indicated that circulating GNBP1 was at a low level in hemolymph (**Figure 3B** **and S4**) despite the presence of signal peptide at N-terminal sequence. To investigate the differential expression of *gnbp3* in translation level, we used a sandwich enzyme-linked immunosorbent assay (ELISA) to detect circulating GNBP3 by coating antibodies against *C*-terminal fragment to the plate and labeling antibodies against *N*-terminal fragment with HRP. ELISA demonstrated that GNBP3 circulated at higher levels in the hemolymph of gregarious locusts (**Figure 3C**).

### Locust GNBP1 responds to pathogen infection

**Figure 4:**
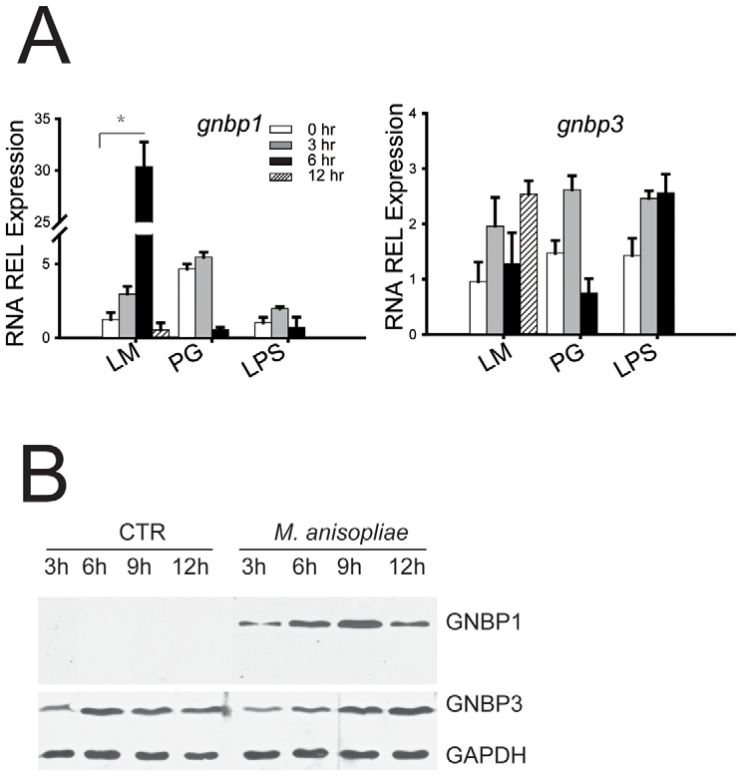
Locust GNBP1 responded to *M. anisopliae* invasion. (A) mRNA expression of locust GNBPs in fat body in respond to injected pathogen associated molecular patterns (PAMPs) were analyzed by quantitative real time PCR. LM: laminarin; PG: peptidoglycan; LPS: lipopolysaccharide. Data was presented as mean (±SE) (B) Immunoblot analysis of GNBPs expression in response to injected conidia (*M. anisopliae*) fat body. CTR: locust saline; fungi: conidia of *M. anisopliae*.

To discern the roles of locust GNBPs in response to *M. anisopliae* infections, we performed immunoblotting assays to determine the distribution of locust GNBPs in immune tissues. GNBP1 was only detected in hemocytes, and was almost undetectable in hemolymph, fat bodies, and midgut. However, GNBP3 was constitutively expressed in most immune tissues such as fat bodies, midgut, hemocytes, and hemolymph (**Figure S4**). Following the injection of pathogen-associated molecular patterns (PAMPs) laminarin (mostly β-1,3-glucan for simulating fungal infection) into hemocoel for 3 h to12 h, the expression of *gnbp1* remarkably increased around 30-fold in fat bodies, but injection of lipopolysaccharide or peptidoglycans (LPS) only slightly altered the expression of *gnbp1* (**Figure 4A** **, left panel**). Surprisingly, *gnbp3* with a high level of expression in fat bodies showed little response to the PAMPs injections (laminarin, peptidoglycan or LPS) (**Figure 4A** **, right panel**). After fungal conidia injection, the GNBP1 in the fat body increased remarkably after 6 h to 9 h, but GNBP3 expression responded minimally to the conidia injection (**Figure 4B**). Moreover, we found circulating GNBP1 in hemolymph was also induced by conidia injection but this was not seen with GNBP3 (**Figure S5**).

### Locust GNBP3 involved in triggering intracellular responses and proteolytic susceptibility

**Figure 5:**
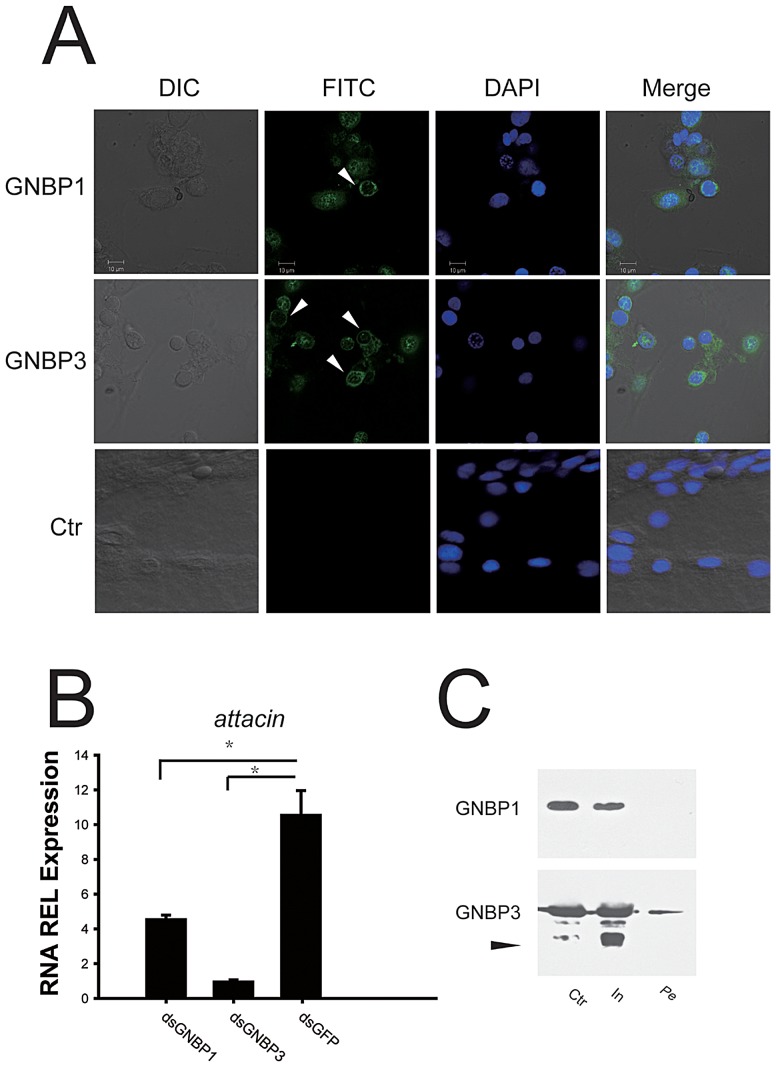
Locust GNBP3 affected *attacin* expression and was susceptible to humoral protease. (A) Immunofluorescence observed the locust GNBPs binding to fungi cell wall by confocal microscope. DIC: differential interference contrast; FITC: fluoresceinisothiocyanate staining; DAPI: 4′,6-diamidino-2-phenylindole staining; Ctr: *Drosophila* hemolymph injected with *M. anisopliae* was used as negative control for examining reactivity of anti-GNBP1 and anti-GNBP3 pAbs. White arrow indicated positive signals. (B) Locust *attacin* expression in fat body after RNAi knockdown of GNBP1 or GNBP3 was examined by quantitative real time PCR. Fold change was normalized to GFP knockdown locusts. (Mann-Whitney U test, n_GNBP1_ = 9, P<0.001; n_GNBP3_ = 9, P<0.001) (C) After 2-hours incubation of laminarin-stimulated hemolymph at room temperature, the proteolysis samples of GNBP1 and GNBP3 proteins in supernatant and clot were analyzed by immunoblot. Ctr: before incubation; In: 2-hours incubation; Pe: pellets after incubation. The black arrow indicates the degraded fragment of GNBP3 recognized by anti-N-terminal pAbs.

Through the use of an immunofluorescence assay we were able to demonstrate that both GNBP1 and GNBP3 in locust hemolymph are able to bind conidial cell wall (**Figure 5A**). Moreover, Locust *attacin*, that dramatically responds to peptidoglycan injection, was also induced by fungus-associated molecular pattern (laminarin, mostly β-1,3-glucan) (**Figure S6**). We successfully knocked down GNBPs protein expression by RNAi method (**Figure S7**), which allowed us to observe their effect on *attacin* expression. The transcriptional level of *attacin* was significantly reduced by *gnbp3* knockdown (n = 12, *P*<0.001, Mann-Whitney U test); however, *gnbp1* knockdown also suppressed the transcriptional level of *attacin* (n = 12, P<0.001, Mann-Whitney U test) (**Figure 5B**). These results showed that circulating GNBP3 was crucial for the activation of *attacin* transcription induced by laminarin. Interestingly, GNBP1 affected *attacin* expression through as yet unknown mechanisms.

Furthermore, the alignment of amino acid sequences showed that a proline-rich sequence (total 32 amino acid residues comprising11 proline residues which commonly acts as a “stick arm” for protein-protein interactions) was inserted into two domains (CRD and GH16) of GNBP3 (**Figure S8**) [34]. These proline-rich sequences are also inserted into the GNBPs of Bm-GNBP3, Bm-GNBP1 (*Bombyx mori*) and Dm-GNBP3 (*D. melanogaster*), except for inducible GNBPs, such as Dm-GNBP2 and Bm-GNBP2, which have shorter inserted sequences and fewer proline residues (**Figure S9**). Locust GNBP1, but not GNBP3, retained an *N*-terminal fragment after humoral protease treatment (**Figure 5C**). Thus, the proteolysis susceptible GNBP3 increased the instability of assembled GNBP3-complex. After laminarin challenge, knocking down of *gnbp3* expression led to a non-detectable melanization in hemolymph (data not shown), indicating that GNBP3 is essential for the initiation of melanization.

### Locust GNBP3 mediates the phenotypic defenses against *M. anisopliae* infections

**Figure 6:**
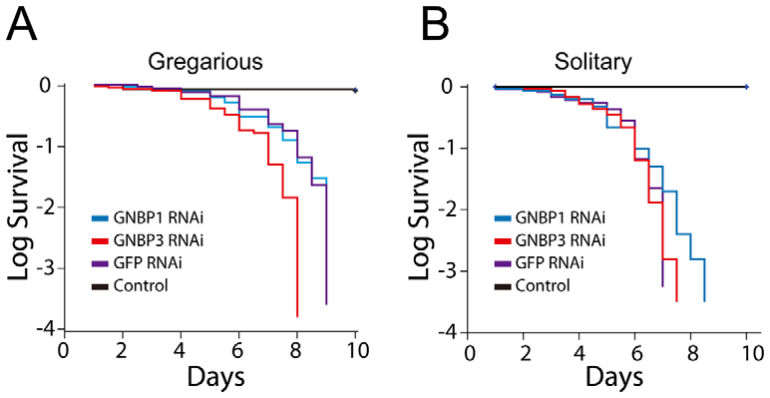
Knockdown GNBP3 affected the locust phenotypic resistance to *M. anisopliae.* Adult male locusts of two phase were inoculated by *M. anisopliae* without septic injury under protonum after knockdown *gnbp1* and *gnbp3* expression for 48 hours by injection of dsRNA. (A) Log survival curves of gregarious locusts after GNBP1 and GNBP3 knockdown by dsRNAi injection (n_GNBP1_ = 36, n_GNBP3 = _44, n_GFP = _32, n_control_ = 27; *P*_GNBP1 vs. GFP_ = 0.796, *P*_GNBP3 vs GFP_ = 0.006). (B) Log survival curves of solitary locusts after GNBP1 and GNBP3 knockdown (n_GNBP1_ = 33, n_GNBP3_ = 30, n_GFP = _22, n_control_ = 27; *P*_GNBP1 vs. GFP = _0.389, *P*_GNBP3 vs. GFP = _0.941). Animals injected with dsGFP for infection were used as negative control; animals without RNAi knockdown and *M. anisopliae* infection were used as naïve group. Kaplan-Meier method in SPSS 13.0 was used to analysis locust survival data.

Insects' innate immune responses to fungal infection are activated by GNBPs and PSH proteolysis pathways [28]. In our transcriptome and molecular functional analysis, prophylactically driven expression of GNBP3 in gregarious locusts indicated that GNBP3-dependent pathway could have a crucial impact on locust phenotypic resistance to fungal infection. When adult male locusts were injected with 20 µg of dsRNA that targeted *gnbp*s, the expression levels of *gnbp1* and *gnbp3* were reduced to around 6% and 15%, respectively, after 48 h (**Figure S10**). Knockdown of *gnbp3* in gregarious locusts significantly reduced the mean survival time to 6.0±0.3 days compared with GFP RNAi control (7.0±0.2 days) (χ^2 = ^7.480, *P_GNBP3 vs. GFP = _*0.006, Log-rank test). Knockdown of *gnbp1* in gregarious locusts did not cause significant changes in life span (χ^2^ = 0.67, *P _GNBP1 vs. GFP_*_ = _0.796) (**Figure 6A**). Moreover, silencing of *gnbp1* or *gnbp3* in solitary locusts did not significantly affect their life spans (χ^2^ = 0.743, *P_GNBP1_*_ vs. GFP_ = 0.389; χ^2 = ^0.076, *P_GNBP3 vs. GFP_ = *0.783) (**Figure 6B**). The significant extension of the life span in gregarious locust but not in solitary locusts indicated that the GNBP3-dependent immune defenses are involved in the phenotypic resistance of locust to *M.anisopliae*.

## Discussion

**Figure 7:**
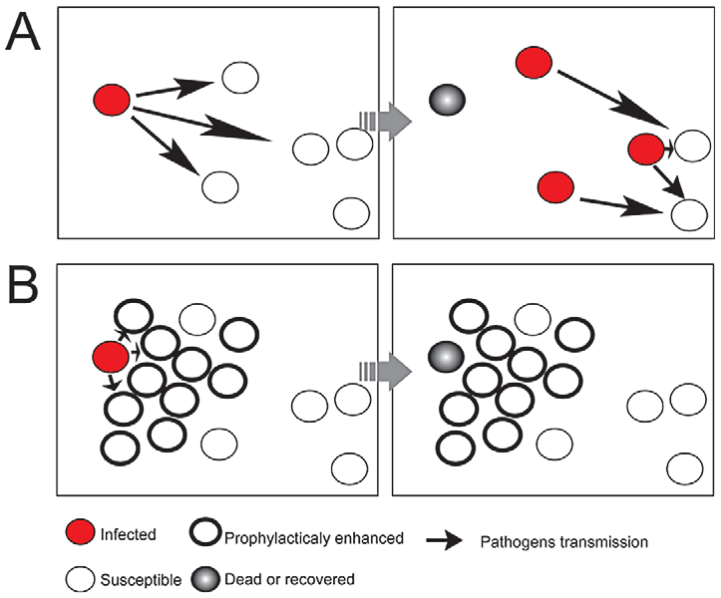
A model for prophylactic immunity in suppression of pathogens spread. A) Pathogens spread through entire population from the infective individuals to susceptible without any obstacles; B) The clustered individuals applied prophylactic immunity to increase the “distance” between the susceptible and infective individuals, as well as the probability of stochastic extinction of the pathogens.

The large scale devastation of crops by locusts is largely the result of formation of gregarious phase by migratory locust in response to high population density. Moreover, the prophylactic immunity of gregarious locusts reduces the susceptibility to fungal biopesticide. Our comprehensive transcriptome analysis revealed that gregarious migratory locusts selectively increased molecules of immune cascades including PRPs, inhibitors (serpins) and counteragents (peroxiredoxin) of ROS rather than activate the entire immune pathways to produce specific effectors (e.g. AMPs), improving our understanding of the molecular dynamics in insect prophylactic immunity. The benefit of emphasizing on the upstream of immune cascades could be to deposit sufficient products of immune responses (eg. melanin) onto the surface of pathogens whilst conserving resources for the production of specific effectors for migration. This defense strategy of coating pathogens to decrease their ability to obtain nutrition from the insect hemolymph leads to an inhibition of pathogens proliferation and production of infective particles. Hence, we suggested that a high level of PRP (GNBP3) allowed gregarious locusts to adapt a tolerance strategy [14], [15] of quarantining the fungal pathogens rather than through their direct elimination. The employment of this strategy reduced the output of *M. anisopliae* conidia from infectious individuals, increased the probability of stochastic extinction, presumably by increasing the “distance” between infected and healthy individuals (**Figure 7**), which has been proposed as an explanation for density dependent prophylaxis [7] based on the Watve and Jog's model [8]. This effect of prophylactic immunity possessed by gregarious locusts is highly similar to an “immunized” status in the herd immunity.

We found that a proline-rich sequence in locust GNBP3, involved in activating fungal defenses, was widely expressed in other species (**Figure S9**). Despite little knowledge about proline-rich sequences in hemolymph proteins recruitment [17], [34], insect GNBP3 was proven to activate humoral and cellular immune responses with the ability to specifically recognize and bind to pathogens in a manner similar to mammalian antibodies. Therefore, due to the versatile roles in immune defenses [17], [35], [36], [37], insect pattern recognition proteins are considered to be good indicators for the assessment of immune defense. Interestingly, GNBP3 assembled attack complexes on fungal pathogens were unstable because of their susceptibility to proteolysis (**Figure 5C**), this resulted in the exposure of fungal PAMPs and caused the recurrent immune activation on the surface of *M. anisopliae*. However, GNBP1 induced by the PAMPs was resistant to proteolysis and therefore is likely to attenuate immune cascades by shielding PAMPs with stable GNBP1-PAMPs complexes, preventing the overstimulation of the immune response in locusts.

After *M. anisopliae* infection, solitary migratory locusts increased the expression of immune genes as well as behavioral genes (eg. chemosensory protein (CSP), juvenile hormone (JH) metabolic enzymes and neuroparsin) that play important roles in migratory locust gregarization [38]. We also found that the basal level of these genes in gregarious locusts was higher than that in solitary locusts (**Data S1**). Moreover, previous studies have found that gregarious desert locusts had an enhanced behavioral fever to resist *M. anisopliae* infection [3], [39]. These results implied that investment into behavioral defenses were part of prophylactic immunity in the arms race between host and pathogens. Thus, more investigations on behavioral defenses at genetic level are required to interpret immune prophylaxis in non-social species, such as studies have the potential to provide the clues to understanding social prophylaxis in eusocial insect immunity [40], [41], [42], [43], [44].

Prophylaxis immunity has been observed in various species and could provide the answer to improved resistance to pathogens at high population densities. However, previous paradoxical observations from restricted investigations limit our understanding of density dependent prophylactic immunity, and have resulted in the polarized sentiments between physiological and ecological immunologist [45]. With the genome wide analysis of locust transcripts, here we found that gregarious migratory locusts presented high levels of circulating PRPs but not AMPs, which suggested a selection of a tolerance strategy for inhibiting pathogen spread and for increasing the “distance” between infected and susceptible individuals that together improved the immune defense of gregarious locusts. This has obvious implications for managing insect pests with high population densities by using entomopathogens-based bio-pesticides, and can help us better understand the prophylaxis immunity in host adaptation of parasites as well as disease epidemics in crowded populations.

## Materials and Methods

### Animals and chemicals

The migratory locusts were collected from North China plain (Huanghua, Hebei Province) and gregarious locust model were established by rearing in large, well-ventilated, cages (25 cm×25 cm×25 cm) at densities of 200 to 300 insects per cage for more than 10 generations. A solitary locust model was established by rearing gregarious locust under physical, visual and olfactory isolation. These conditions were achieved by using a ventilated cage (10 cm×10 cm×25 cm) with charcoal-filtered compressed air for more than 10 generations, which maintained the phase-traits for reversible phase transition. Both gregarious and solitary cultures were reared under a 14∶10 light/dark photo regime at 30±2°C and on a diet of fresh greenhouse-grown wheat seedlings and wheat bran [18], [46]. The adult locusts were analyzed at 3 d to 5 d after molting when male and female nymphs were previously separated at the end of 5th instar period to avoid mating. Peptidoglycans, lipopolysaccharides (LPS) and laminarin were injected at a dose of 20, 80, and 100 µg/insect, respectively, according to previous studies [47], [48], [49]. The chemicals were purchased from Sigma unless otherwise indicated.

### Survival analysis

At 4 d after molting, locust individuals were inoculated via standard procedures as previously described [50]. Briefly, the locusts from each phase were inoculated with 2 µL peanut oil (Sigma, USA) containing 1×10^6^ conidia (IMP003, *M. anisoplia*e [51]) under the pronotum. This method is noninvasive, and the peanut oil control has negligible effects on mortality [52]. The locusts were maintained in individual containers under a 12 h∶12 h light: dark circle at 22°C to 25°C, and were fed and assessed for mortality twice daily. The survival curves were compared using Kaplan-Meier and Cox's proportional hazards model was used for assessing variables that affect locusts survival. The threshold of P value was adjusted by Bonferroni correction. SPSS 13.0 software was used in all statistical analyze.

### RNA-sequencing transcriptome experiments

To investigate the prophylactic immunity and responses to *M. ansopliae* infection in the two locust phases, we used high throughput sequencing (HTS) platform (HiSeq 2000) to analysis genes expression in pre- and post-fungal infected gregarious and solitary locust. At 6 d to 7 d (TL_50_ of solitary and gregarious locust respectively) after inoculation of 1×10^6^ conidia (*M. anisopliae*) or peanut oil (mean life of solitary or gregarious locusts, respectively), three replicates for each samples (25 individuals/sample) were pooled for analysis (GC: gregarious locust control, GI: gregarious locust infected, SC: solitary locust control, and SI: solitary locust infected). 8 µg of total mRNA from each sample was used to construct libraries with an Illumina kit v2. After paired-end sequencing, the raw reads were assembled by Trinity software (version 2011-08-20) to obtain reference transcripts due to no published locust genome data. Downstream analysis of alignment (Bowtie) and abundance estimation (RSEM) were performed using the utility package in Trinity software (version 2011-08-20). The differential expressed transcripts were analyzed using DEseq and EdgeR software [53], [54]. Finally, Blast2Go was used to annotate and enrich the DETs (P<10^−6^) [55]. The raw reads of 4 samples are available for download form the NCBI SRA server (accession number: SRA054168).

### Sample preparation of fat bodies, midgut, hemolymph, and hemocytes

Hemolymph was immediately collected in 1 mL of ice-cold saline and then centrifuged at 1000× g at 4°C for 3 min to separate the hemocytes, which were washed thrice with 1 mL of locust saline. The insect tissues (fat body and midgut) were dissected immediately after hemolymph collection. All samples were directly frozen in liquid nitrogen until RNA and protein sample preparation. RNA was extracted using Trizol (Invitrogen, USA) according to the manufacturer's instructions. Proteins were sequentially extracted in Trizol according to the manufacturer's instructions. The protein pellets were weighed and completely dissolved overnight at 100 µg/µL in loading buffer containing 2 M thiourea, 7 M urea, 1% sodium dodecyl sulfate (SDS), 1% dithiothreitol (DTT), and 100 mM Tris HCl (pH 7.0) (rehydration buffer for two-dimensional electrophoresis, Bio-Rad). Cell-free protein concentration of hemolymph was measured with the bicinchoninic acid (BCA) method (Pierce, USA).

### Rapid amplification of cDNA ends (RACE)

RACE experiments were performed to obtain the full-length of cDNAs (*gnbp1*, *gnbp2*, and *gnbp3*) to examine the RNA-seq assembly quality. A BD SMART RACE cDNA Amplification Kit was used to amplify the 5′ - and 3′ -ends of locust Gram-negative bacteria-binding protein (*gnbp*) cDNAs according to the manufacturer's protocol. The fragment of the target sequence was assembled using CONTIGEXPRESS software. Finally, the sequence was verified by sequencing the full-length PCR amplification products inserted into the pGEM-T Easy vector.

### Bioinformatisc analysis

To observe locust *gnbp* family classification, the derived protein sequences of GNBPs were identified using a BLAST search against other species. All GNBP sequences (without signal sequences) were aligned using ClustalX 1.83, and the alignment data was used to construct phylogenetic tree with MEGA 4.0. The tree was rooted on *B. circulans* β-(1,3)-glucanase (AAC60453). One thousand replicates were used to calculate the bootstrap values, and branches were collapsed with a 50% consensus rule.

### Real-time quantitative PCR (qPCR) analysis

To observe target genes expression, qPCR experiments were performed according to the standard protocols for thermocyclers (Stratagene and Roche, USA). Four biological replicates (3 to 5 individuals per replicate) were pooled for three parallel technical replicates analysis. The mRNA of each sample was extracted from dissected tissues using Trizol. The ratios of OD_260_/OD_280_ were then measured. The cDNA was synthesized from 2 µg of total RNA with MLV reverse II system (Promega, USA). The primers were designed based on PRIMER 5.0 (also see Table S6). The *actin* sequence of *L*. *migratoria* (GenBank accession no: AF370793) were used for internal control and the target gene sequences cloned into pGEM-T Easy for standard curve calibration. The specificity of amplification was confirmed through melting curve analysis. Values were represented as the mean (±SE), and the statistical significance was determined by using Mann-Whitney U test with SPSS 13.0 software.

### Antibody preparation

To obtain the detectors for GNBPs proteins, we recombinantly expressed the highly antigenic fragments of the locust GNBPs. The antigenicity of the proteins was analyzed with an online tool (http://imed.med.ucm.es/Tools/antigenic.pl). The recombinant proteins that contained the highly antigenic truncated sequences (GNBP1 JF915523 ORF: N terminal 125–808 bp, C-terminal 1103–1411 bp; GNBP3 JF915525 ORF: 95–901 bp) were expressed using the Invitrogen pET-28a vector in *E. coli.* The proteins were then purified with Ni-Sepharose media (GE Healthcare, USA). After 12 weeks of immunization, the rabbit polyclonal antibodies sera were purified by amino sulfate precipitation and tested for specificity using the recombinant full-length GNBPs from SF9 cells. The cross-reactive antibodies were absorbed by immobilized rGNBPs (SF9 cell-expressed) on CNBr-activated Sepharose 4B (GE Healthcare, USA).

### Expression of GNBPs in SF9 cells

To obtain positive control of ELISA and immunoblotting assay, GNBPs expressed in SF9 cell culture supernatant were used to test antibody cross reactivity. The *gnbp*s, including their signal sequences, were cloned into pFastbac, and were shuttled into DH10bac bacteria. The plasmids were transfected into SF9 cells according to the manufacturer's instructions (Invitrogen, USA). After three rounds of infection, a 2.4×10^7^ pfu/mL titer of virus was added to 1 L SF9 cell culture for infection (Multiplicity of Infection, MOI = 1.2). The secreted GNBP proteins in the supernatant liquid were collected at time points of 48, 72 and 96 hours. The cell debris were removed by centrifugation at 12,000× g for 5 min, and the samples were concentrated 10-fold and buffer-exchanged into locust saline by centrifuging at 18,000× g for 15 min and at 4°C in an Ultra-15 tube (Millipore, USA).

### dsRNAi experiments

To reduce the GNBPs proteins level in locust, we used RNAi method to knockdown the *gnbp*s expression. Templates for dsRNA preparation were PCR-derived fragments between two T7 promoter sequences. The fragments of each gene were: *gfp* (nucleotides ORF35-736, GenBank accession L29345), *gnbp1* (ORF22-437, GenBank accession JF915523), and *gnbp3* (ORF38-534, GenBank accession JF915525). The single-stranded RNA fragments were synthesized using a T7 transcription kit (Promega, USA). The annealed dsRNA were purified by ethanol precipitation then were dissolved in sterilized locust saline buffer at 5 µg/µL until use. Finally, each insect was injected with 20 µg of the dsRNA for experiments.

### Cell staining

To investigate the locust GNBPs capability of binding to fungi cell wall, adult locusts' hemolymph was collected after injected conidia 9 hours. Hemocytes and injected conidia were dispersed on poly-lysine-coated glass slides and then fixed with 1% PEG-8000 in 95% ethanol for 45 min. Cells were then permeabilized with 0.1% Triton X-100, 1% bovine serum albumin and phosphate-buffered saline (PBS) for 1 hour, following blocked with TBS containing 5% skim milk for 4 hours at room temperature. The samples were incubated overnight with fluorescein isothiocyanate (FITC)-conjugated polyclonal antibodies with 400-fold dilution in TBS containing 0.1% Tween-20 and 5% skim milk. After staining with 4′6-diamidino-2-phenylindole (DAPI) in PBS for 15 min, the samples were washed five times and sealed in 50% glycerol in PBS for observation under a confocal microscope (LSM710, Carl Zeiss).

### Immunoblot analysis

To observe locust GNBPs proteins expression, protein samples were prepared using Trizol after mRNA extraction according to the manufacturer's instructions. The samples (each lane for 6 individuals and loaded 10 µg/lane) were loaded, separated using sodium dodecyl sulfate polyacrylamide gel electrophoresis (SDS-PAGE), and transferred onto polyvinylidene difluoride (PVDF) membranes (Bio-Rad, USA). Membranes were blocked in Tris-buffered saline with 5%Tween-20 (TTBS) containing 5% slim milk for 4 hours at room temperature, then probed with primary antibodies (1∶4,000 dilution) at 4°C overnight, washed 3×10 minutes in TTBS, incubated for 1 hour with secondary antibodies (HRP-conjugated mouse anti-rabbit monoclonal antibody; Sigma A1949, USA) at room temperature, and visualized under an enhanced chemiluminescence system (Bio-Rad, USA) or recorded on X-ray films (Kodak, USA). We used locust glyceraldehyde-3-phosphate dehydrogenase (GAPDH) for internal control of proteins amounts. The polyclonal antibodies against locust GNBP1, GNBP3 and GAPDH were previously purified and stored in PBS (containing 50% glycerol) at −80°C until use.

### Enzyme-linked immunosorbent assay (ELISA)

To investigate the locust circulating GNBPs in hemolymph, polyclonal antibodies against the *C*-terminal of GNBP1 was dissolved in pH 9.5 carbonate buffers at a concentration of 500 ng/mL and coated onto plates (Costar 92592, USA) overnight at 100 µL/well. The plates were washed three times with TTBS, incubated with 200 µL/well of 20% fetal calf serum at 37°C for 2 h, and then washed 3 times with TTBS. Samples with same total proteins amounts (gregarious: n = 9 and solitarious: n = 15) were applied at 100 µL/well and incubated at 37°C for 1 hours. Finally, plates were washed three times with TTBS, incubated with 100 µL/well of horse radish peroxidase (HRP) conjugated polyclonal antibodies against *N*-terminal of GNBPs (1∶1000 dilution) to detect the positive signals. The reaction was stopped with 50 µL/well of 2 mol/L sulfuric acid. The OD_450_ values were recorded for further statistical analysis.

### Proteolysis assay

To examine locust GNBPs resistance to hemolymph protease, the hemolymph from laminarin-stimulated locusts (n = 12) was collected and immediately diluted tenfold with ice-cold saline. Hemocytes were removed by centrifugation at 1,000× g for 3 minutes, and hemolymph was incubated at 25°C for 1 h. The incubated samples were centrifuged at 12,000× g for 20 minutes to collect the supernatant liquid and clot pellets. All samples were denatured, and were then loaded into gels (10 µg/lane) for SDS-PAGE separation. After electrophoresis, the proteins in gels were transferred onto a PVDF membrane for immunoblot analysis. The primary polyclonal antibodies against GNBPs *C*-terminal fragment were applied at 1∶3,000 dilution, and the secondary monoclonal antibody (A1949, Sigma, USA) were applied at 1∶12,000 to detect target proteins through chemiluminescence.

## Supporting Information

Data S1 **Up regulated differentially expressed transcripts of pre- and post-infected fatbody samples of the two phase locusts.** Behavioral and immune cascade-related differentially expressed transcripts were calculated by DEGseq software as a cut off (*P*<0.001, Q-value<0.05). GC: fat body sample of pre-infected gregarious locusts; GI: fat body sample of gregarious locusts infected by fungi *M. anisopliae*; SC: fat body sample of pre-infected solitary locusts; SI: fat body sample of solitary locusts infected by fungi *M. anisopliae.*
(XLS)

Figure S1 **Differentially expressed transcripts were analyzed by DEGseq software.** M-A plot of DEGseq displayed the A) differentially expressed transcripts between pre-infected solitary (SC) and gregarious locusts (GC); B) Differentially expressed transcripts between pre- and post- infected solitary locusts (SC, SI); C) Differentially expressed transcripts between pre- and post-infected gregarious locusts (GC, GI); D) Differentially expressed transcripts of *M. anisopliae* infected gregarious locusts and solitary locusts (GI, SI). The numbers in parentheses are the transcripts significantly up- or down-regulated by each treatment of the two phases of locusts.
(TIF)

Figure S2 **Differentially expressed transcripts were analyzed by EdgeR software.** M-A plot of EdgeR displayed the A) differentially expressed transcripts between pre-infected solitary (SC) and gregarious locusts (GC); B) differentially expressed transcripts between pre- and post-infected solitary locusts (SC, SI); C) differentially expressed transcripts between pre- and post-infected gregarious locusts (GC, GI); D) differential expressed transcripts between *M. anisopliae* infected gregarious locusts and solitary locusts (GI, SI). The numbers in parentheses are the transcripts significantly up- or down-regulated by each treatment of the two phases of locusts.
(TIF)

Figure S3 **Bioinformatic analysis of the derived protein sequences of GNBP family.** A) Distance tree of insect GNBPs rooted with *B. circulans* β-1,3-glucanase. Left clade clustered GNBPs with two domains (CRD and GH16), and right clade clustered GNBPs with one domain domain (GH16). Two-domain GNBPs were considered as pattern recognition proteins, and one-domain GNBPs were considered as digestion proteins with glucanase activities. Lm: *Locusta migratoria*; Ag: *Anopheles gambiae*; Bm: *Bombyx mori*; Dm: *Drosophila melanogaster*; Am: *Apis mellifera*; Tc: *Tribolium castaneum*; and Nf: *Nasutitermes fumigates*; B) Schematic protein structure of locust GNBPs family. CRD: Carbohydrate Recognition domain; GH16: Glycoside hydrolase 16.
(TIF)

Figure S4 **Immunoblot analysis of locust GNBPs distribution in immune tissues.** Locust immune tissue samples including hemocytes, hemolymph, fat body and midgut were prepared for examining GNBPs expression. Up panel indicated the immunoblot results of GNBP1 distribution in immune tissues; lower panel indicated the immunoblot results of GNBP3 and internal control GAPDH protein.
(TIF)

Figure S5 **Immunoblot analysis of GNBPs in response to fungi conidia (** ***M. anisopliae*** **) injection in the hemolymph.** Locust hemolymph samples were collected by removing hemocytes then loaded for immunoblot analysis. Up panel indicated immunoblot results of GNBP1 response to conidia challenge in hemolymph; Lower panel indicated immunoblot results of GNBP3 response to conidia challenge in hemolymph; N: Control sample.
(TIF)

Figure S6 **Q-PCR analysis of locust** ***attacin*** **expression in response to pathogen associated molecular patterns.** Locust *attacin* expression in fat body was determined by Q-PCR during 3–24 h after injection of PAMPs. LM: Laminarin; PG: peptidoglycan; LPS: lipopolysaccharide.
(TIF)

Figure S7 **Immunoblot analysis RNAi efficiency of GNBP1 and GNBP3.** A) After injected dsRNAi 4 days, the laminarin was injected into locust hemoceol and collected fat body samples from 3 to 12 hours by 3 hours interval to observe GNBP1 expression. B) Fat body samples after dsRNA injection from 1 day to 4 day were collected and observed GNBP3 expression by immunoblot.
(TIF)

Figure S8 **Protein sequences of locust GNBPs family were aligned to examine their homology levels.** The amino acids shadowed with black color were 100% identity, and >50% homology level were boxed with cyan color. Underlined amino acids with red color line indicated the inserted sequences in GNBP3, and the arrows indicated proline amino acids (11 proline amino acids).
(TIF)

Figure S9 **Align insect GNBPs to observe proline rich sequences between CRD and GH16 domain.** Inserted protein sequences between two domains were boxed with red line; the amino acids shadowed with black color were 100% homology level, >70% homology level were shadowed with magenta color and >50% with cyan color. Lm: *locusta migratoria*; Bm: *Bombyx mori*; Dm: *Drosophila melanogaster*; Pi: *Plodia interpunctella*.
(TIF)

Figure S10 **Q-PCR examination of locust GNBPs RNAi efficiency.** After 48 h of injection locust *gnbp* dsRNA, the fat body samples were collected and examined the *gnbp1* and *gnbp3* mRNA transcription. Fold change were presented as mean ±SE.
(TIF)

Table S1 **Cox regression analysis of variables affecting locust's survival after** ***M. anisopliae*** **infection.** Four variables (treatment, gender, weight and phase) were assessed by cox proportional hazard model analysis in SPSS 13.0 (Backward Stepwise Wald method). The hazard ratio of solitary to gregarious after *M. anisopliae* treatment is around 2.6 times (Exp(B) = 1/0.391).
(DOC)

Table S2 **General features of** ***de novo*** **assembled transcripts by Trinity software.** GC: fat body sample of pre-infected gregarious locusts; GI: fat body sample of gregarious locusts infected by fungi *M. anisopliae*; SC: fat body sample of pre-infected solitary locusts; SI: fat body sample of solitary locusts infected by fungi *M. anisopliae*; shaded sample was selected as reference transcripts.
(DOC)

Table S3 **Differentially expressed transcripts were determined by EdgeR software.** Different thresholds of significance (adjusted P values) were set for observe differentially expressed transcripts between pre- post-infected samples of the two phases locust.
(DOC)

Table S4 **Differentially expressed transcripts were determined by DEGseq software.** Different thresholds of significance (P values) and false discovery rate (FDR, Q-values) were set for observe differentially expressed transcripts between pre- post-infected samples of the two phases locust.
(DOC)

Table S5 **Differentially expressed genes were confirmed by qPCR experiments.** Twelve differentially expressed genes determined by RNAseq were confirmed by Q-PCR and showed positive correlation (Pearson *r* = 0.92).
(DOC)

Table S6 **Primers for Q-PCR and amplification of cDNAs.** Reversed (R) and forward (F) primers for Q-PCR and amplification of cDNAs were listed in the table.
(DOC)
