# Supplementary figures and images for: Altered Immunity in Crowded Locust Reduced Fungal (Metarhizium anisopliae) Pathogenesis
Source: PLoS Pathog. 2013 Jan 10;9(1):e1003102. doi: 10.1371/journal.ppat.1003102 (PMC3542111; doi:10.1371/journal.ppat.1003102)

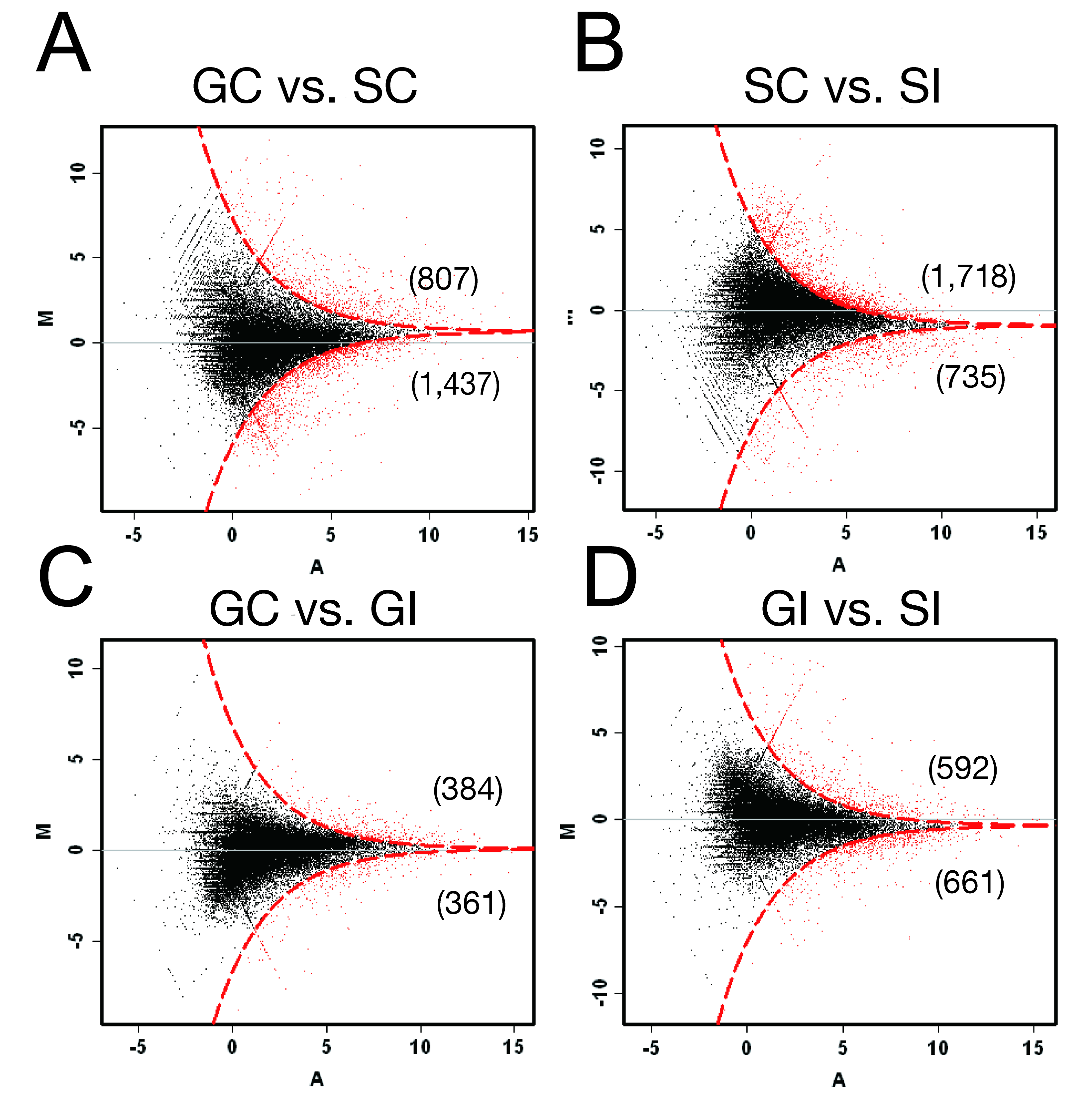

Supplement: Figure S1 — Differentially expressed transcripts were analyzed by DEGseq software. M-A plot of DEGseq displayed the A) differentially expressed transcripts between pre-infected solitary (SC) and gregarious locusts (GC); B) Differentially expressed transcripts between pre- and post- infected solitary locusts (SC, SI); C) Differentially expressed transcripts between pre- and post-infected gregarious locusts (GC, GI); D) Differentially expressed transcripts of M. anisopliae infected gregarious locusts and solitary locusts (GI, SI). The numbers in parentheses are the transcripts significantly up- or down-regulated by each treatment of the two phases of locusts. (TIF) [file ppat.1003102.s002.tif]

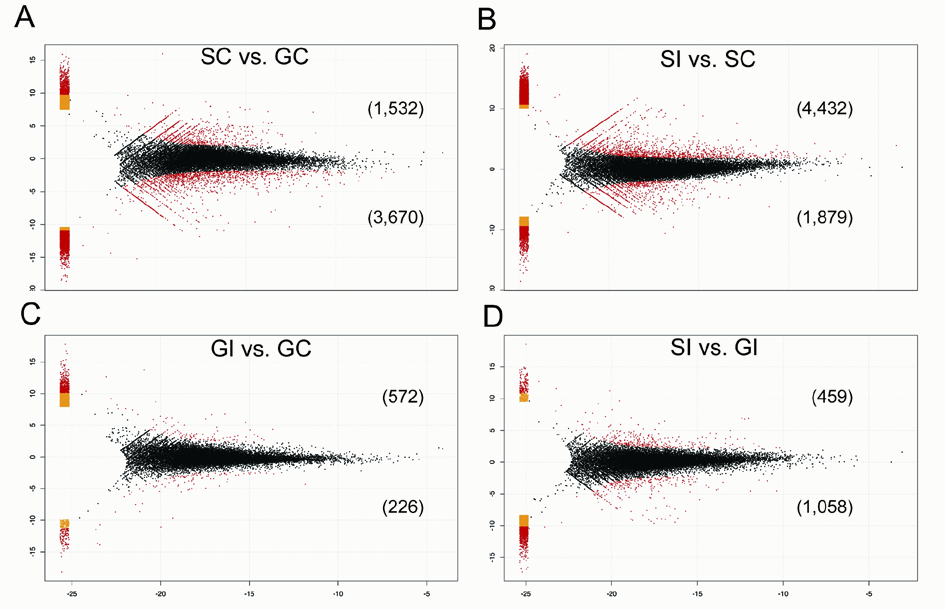

Supplement: Figure S2 — Differentially expressed transcripts were analyzed by EdgeR software. M-A plot of EdgeR displayed the A) differentially expressed transcripts between pre-infected solitary (SC) and gregarious locusts (GC); B) differentially expressed transcripts between pre- and post-infected solitary locusts (SC, SI); C) differentially expressed transcripts between pre- and post-infected gregarious locusts (GC, GI); D) differential expressed transcripts between M. anisopliae infected gregarious locusts and solitary locusts (GI, SI). The numbers in parentheses are the transcripts significantly up- or down-regulated by each treatment of the two phases of locusts. (TIF) [file ppat.1003102.s003.tif]

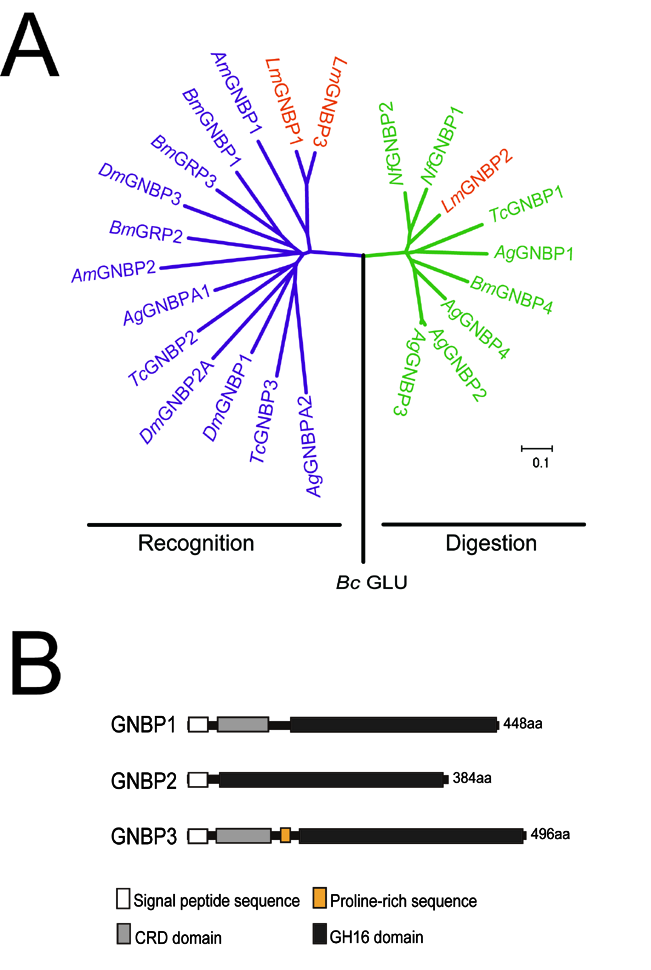

Supplement: Figure S3 — Bioinformatic analysis of the derived protein sequences of GNBP family. A) Distance tree of insect GNBPs rooted with B. circulans β-1,3-glucanase. Left clade clustered GNBPs with two domains (CRD and GH16), and right clade clustered GNBPs with one domain domain (GH16). Two-domain GNBPs were considered as pattern recognition proteins, and one-domain GNBPs were considered as digestion proteins with glucanase activities. Lm: Locusta migratoria; Ag: Anopheles gambiae; Bm: Bombyx mori; Dm: Drosophila melanogaster; Am: Apis mellifera; Tc: Tribolium castaneum; and Nf: Nasutitermes fumigates; B) Schematic protein structure of locust GNBPs family. CRD: Carbohydrate Recognition domain; GH16: Glycoside hydrolase 16. (TIF) [file ppat.1003102.s004.tif]

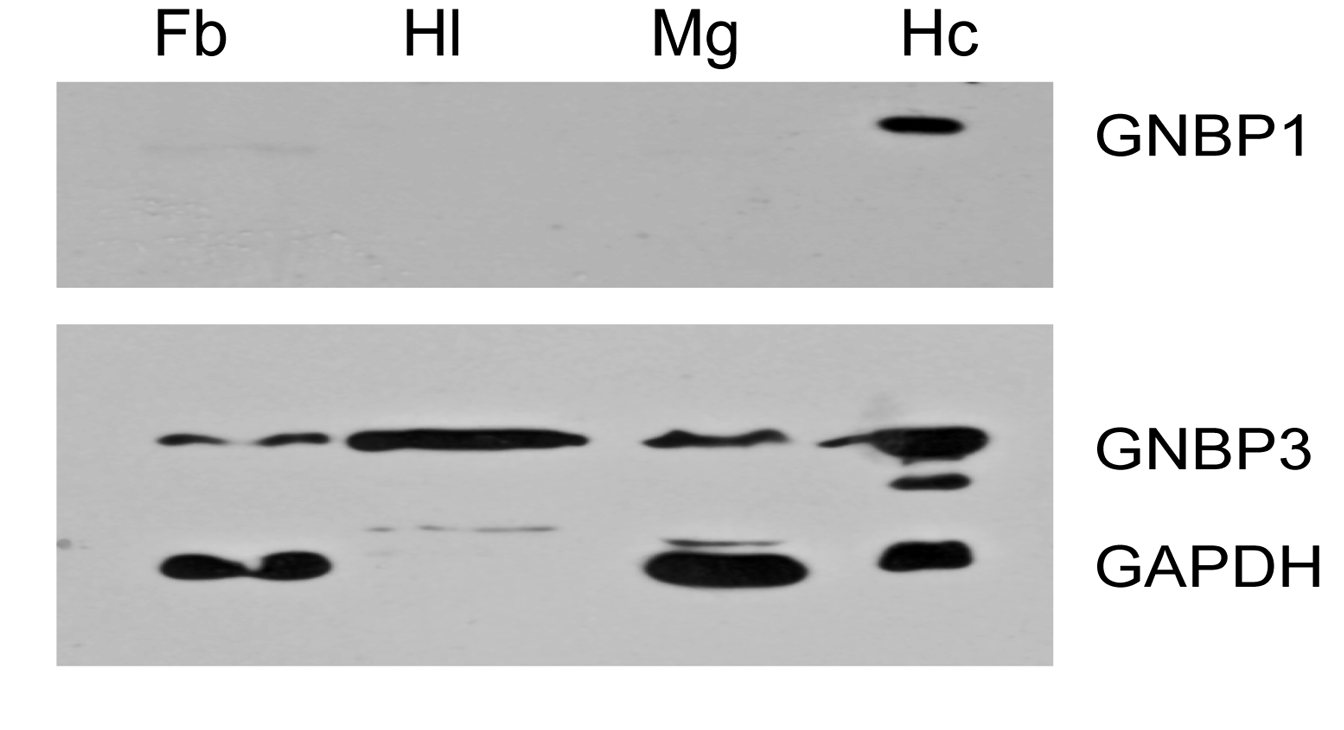

Supplement: Figure S4 — Immunoblot analysis of locust GNBPs distribution in immune tissues. Locust immune tissue samples including hemocytes, hemolymph, fat body and midgut were prepared for examining GNBPs expression. Up panel indicated the immunoblot results of GNBP1 distribution in immune tissues; lower panel indicated the immunoblot results of GNBP3 and internal control GAPDH protein. (TIF) [file ppat.1003102.s005.tif]

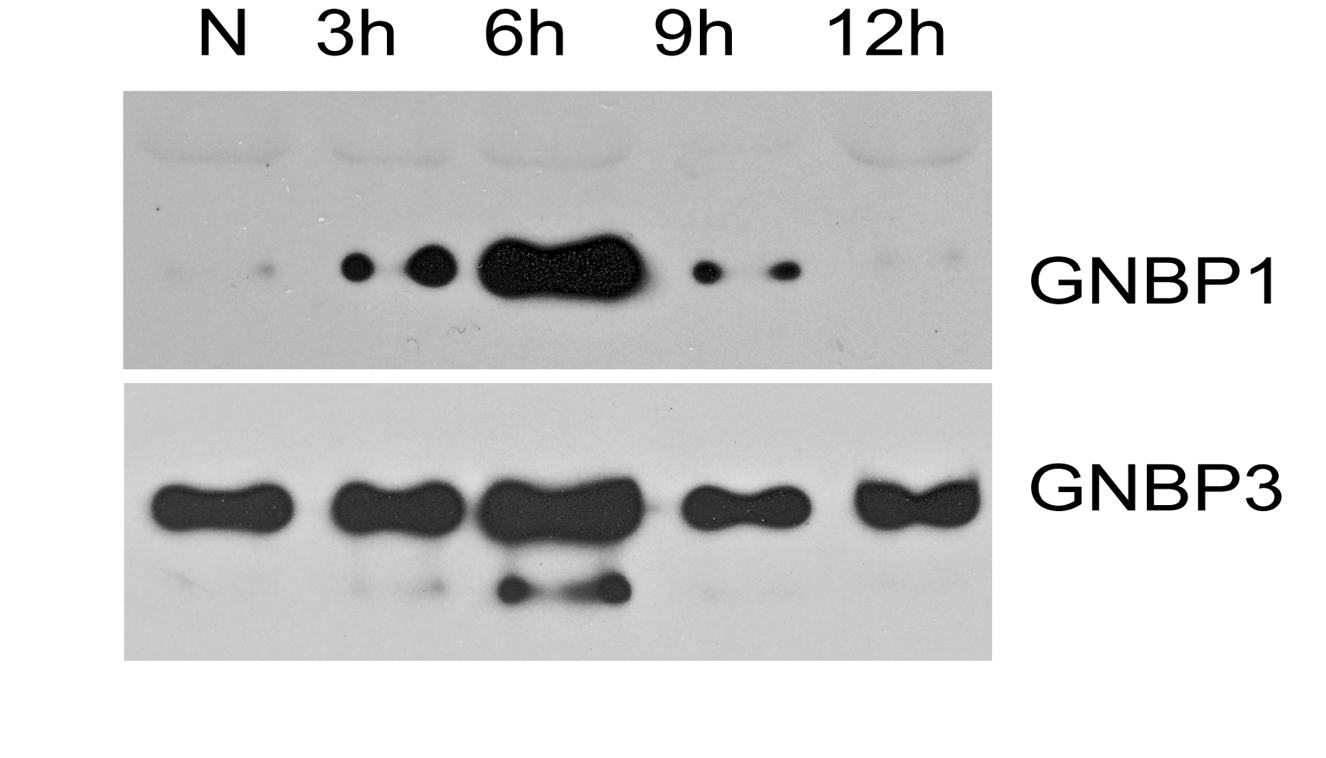

Supplement: Figure S5 — Immunoblot analysis of GNBPs in response to fungi conidia ( M. anisopliae ) injection in the hemolymph. Locust hemolymph samples were collected by removing hemocytes then loaded for immunoblot analysis. Up panel indicated immunoblot results of GNBP1 response to conidia challenge in hemolymph; Lower panel indicated immunoblot results of GNBP3 response to conidia challenge in hemolymph; N: Control sample. (TIF) [file ppat.1003102.s006.tif]

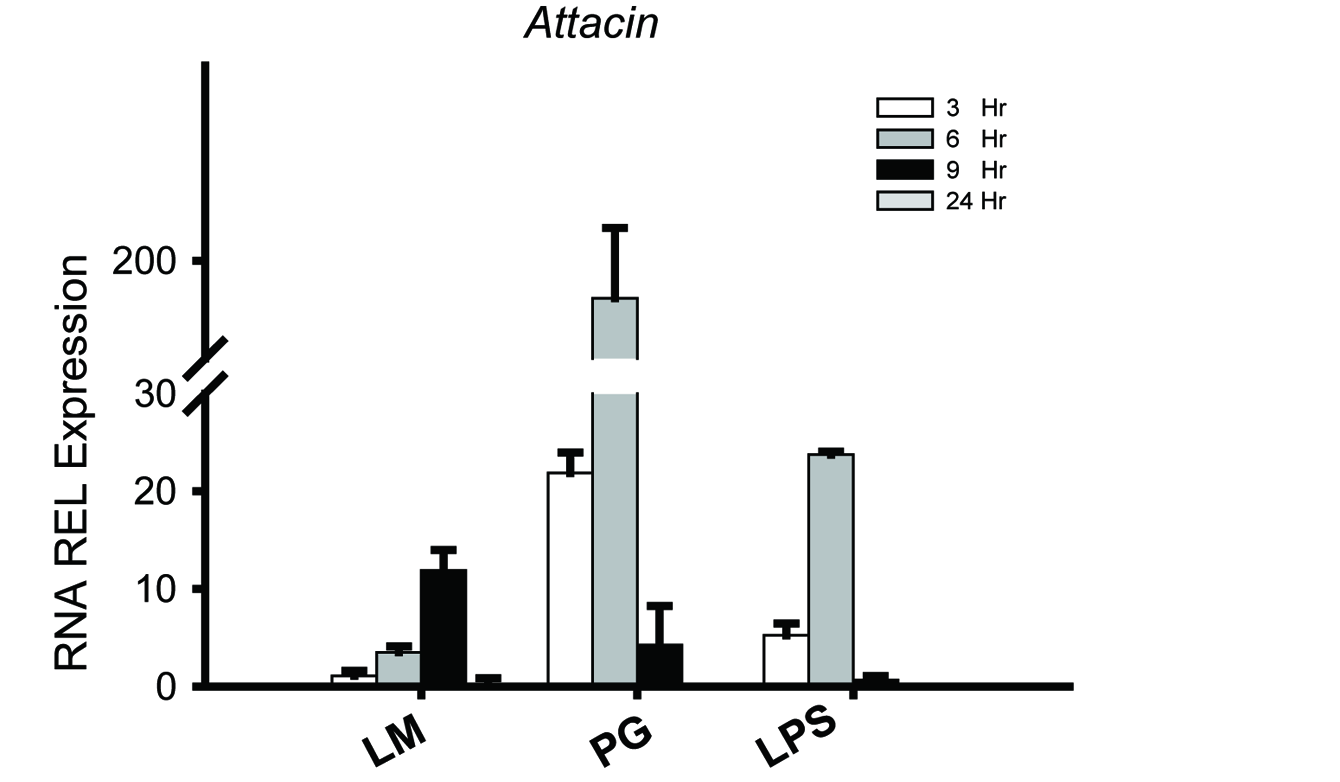

Supplement: Figure S6 — Q-PCR analysis of locust attacin expression in response to pathogen associated molecular patterns. Locust attacin expression in fat body was determined by Q-PCR during 3–24 h after injection of PAMPs. LM: Laminarin; PG: peptidoglycan; LPS: lipopolysaccharide. (TIF) [file ppat.1003102.s007.tif]

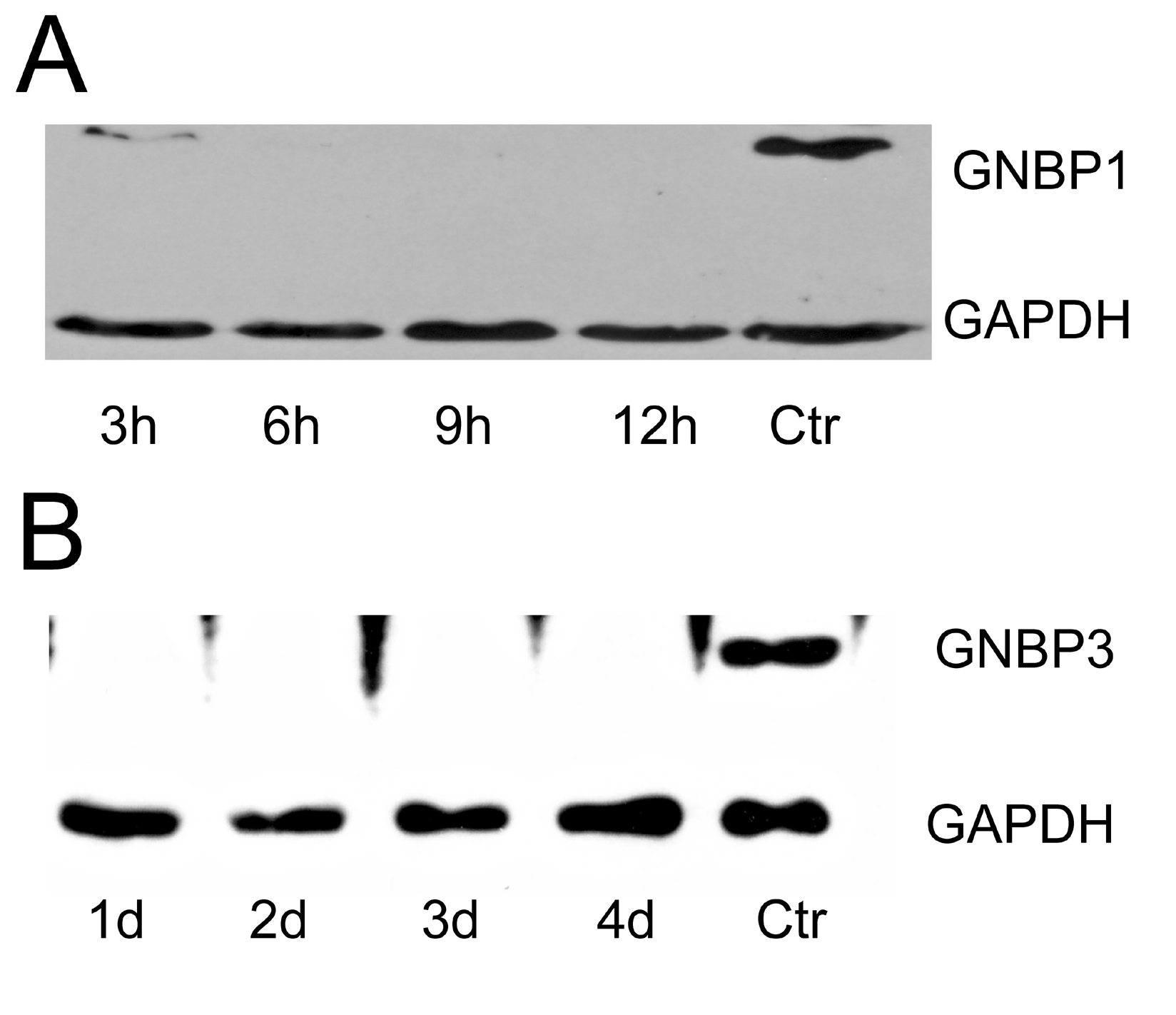

Supplement: Figure S7 — Immunoblot analysis RNAi efficiency of GNBP1 and GNBP3. A) After injected dsRNAi 4 days, the laminarin was injected into locust hemoceol and collected fat body samples from 3 to 12 hours by 3 hours interval to observe GNBP1 expression. B) Fat body samples after dsRNA injection from 1 day to 4 day were collected and observed GNBP3 expression by immunoblot. (TIF) [file ppat.1003102.s008.tif]

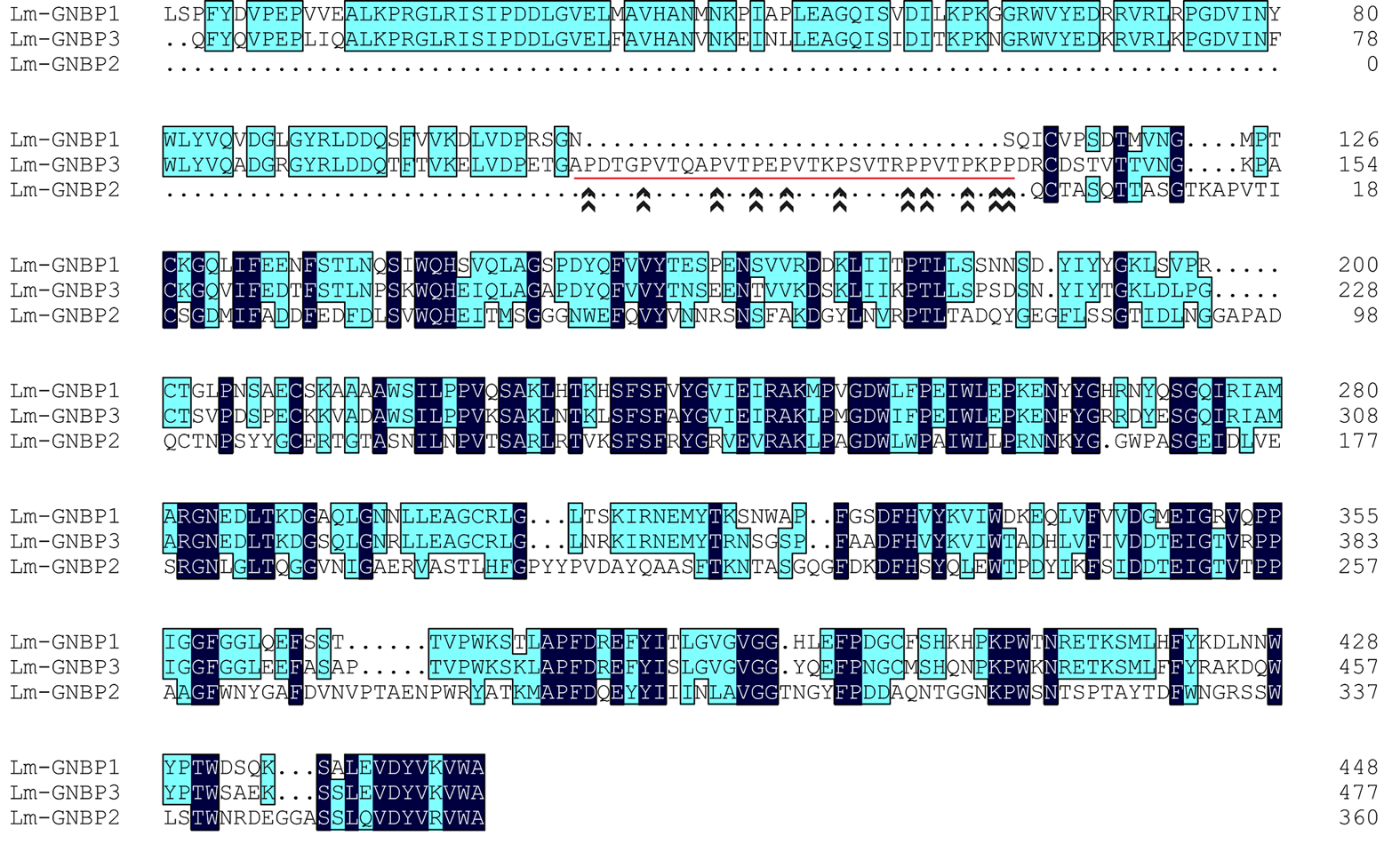

Supplement: Figure S8 — Protein sequences of locust GNBPs family were aligned to examine their homology levels. The amino acids shadowed with black color were 100% identity, and >50% homology level were boxed with cyan color. Underlined amino acids with red color line indicated the inserted sequences in GNBP3, and the arrows indicated proline amino acids (11 proline amino acids). (TIF) [file ppat.1003102.s009.tif]

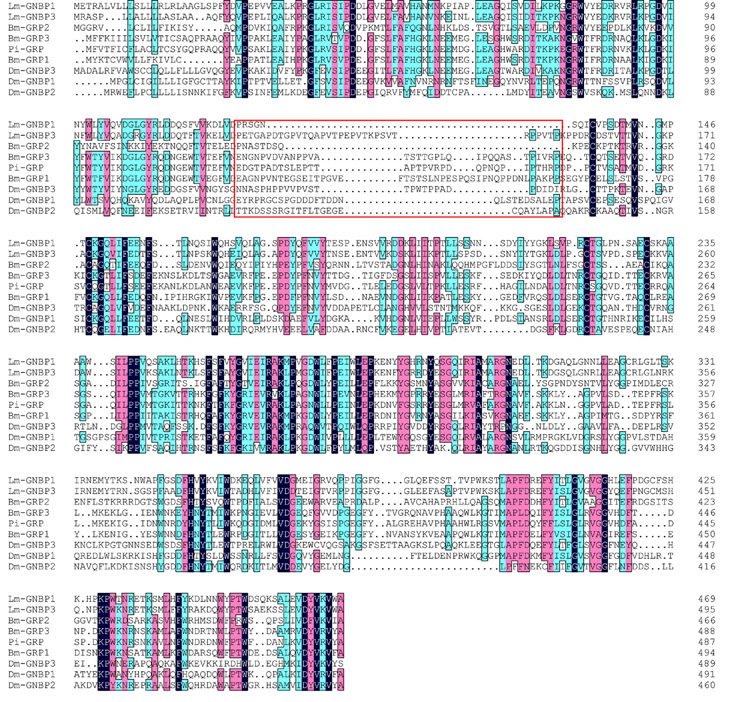

Supplement: Figure S9 — Align insect GNBPs to observe proline rich sequences between CRD and GH16 domain. Inserted protein sequences between two domains were boxed with red line; the amino acids shadowed with black color were 100% homology level, >70% homology level were shadowed with magenta color and >50% with cyan color. Lm: locusta migratoria; Bm: Bombyx mori; Dm: Drosophila melanogaster; Pi: Plodia interpunctella. (TIF) [file ppat.1003102.s010.tif]

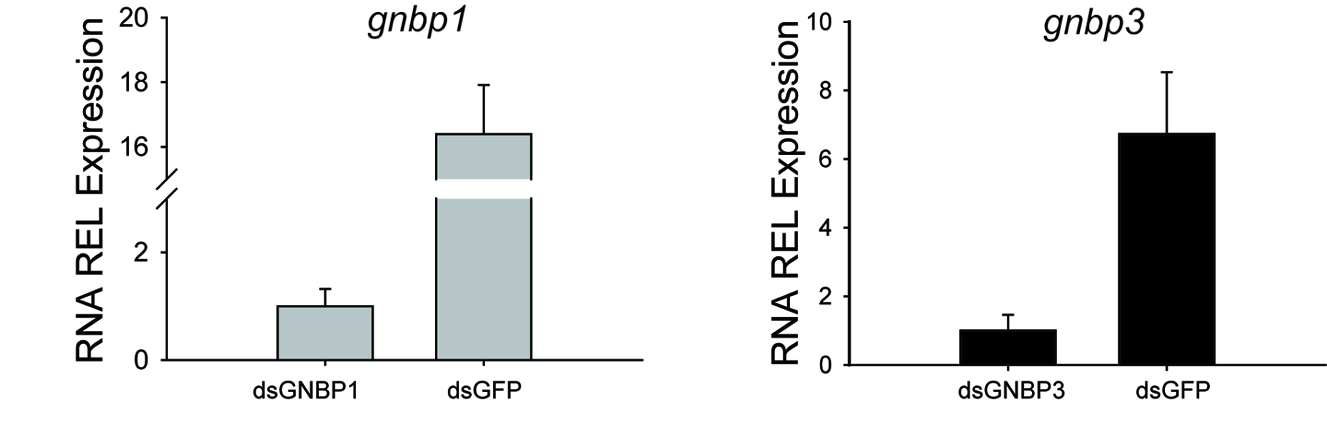

Supplement: Figure S10 — Q-PCR examination of locust GNBPs RNAi efficiency. After 48 h of injection locust gnbp dsRNA, the fat body samples were collected and examined the gnbp1 and gnbp3 mRNA transcription. Fold change were presented as mean ±SE. (TIF) [file ppat.1003102.s011.tif]
