# Supplementary material for: Altered Immunity in Crowded Locust Reduced Fungal (Metarhizium anisopliae) Pathogenesis
Source: PLoS Pathog. 2013 Jan 10;9(1):e1003102. doi: 10.1371/journal.ppat.1003102 (PMC3542111; doi:10.1371/journal.ppat.1003102)
Supplement: Table S1 — Cox regression analysis of variables affecting locust's survival after M. anisopliae infection. Four variables (treatment, gender, weight and phase) were assessed by cox proportional hazard model analysis in SPSS 13.0 (Backward Stepwise Wald method). The hazard ratio of solitary to gregarious after M. anisopliae treatment is around 2.6 times (Exp(B) = 1/0.391). (DOC) [file ppat.1003102.s012.doc]

Table S1 Cox regression analysis of variables affecting locusts survival after *M. anisopliae* infection

|  | |  |  |  |  |  |  | 95.0% CI for Exp(B) | |
| --- | --- | --- | --- | --- | --- | --- | --- | --- | --- |
|  | | B | SE | Wald | df | Sig. | Exp(B) | Lower | Upper |
| Step 1 | Gender | -.430 | .556 | .598 | 1 | .439 | .651 | .219 | 1.933 |
|  | Weight | -.725 | .556 | 1.698 | 1 | .193 | .484 | .163 | 1.441 |
|  | Treat | 5.633 | .628 | 80.579 | 1 | .000 | 279.569 | 81.718 | 956.445 |
|  | Phase | -1.091 | .230 | 22.581 | 1 | .000 | .336 | .214 | .527 |
| Step 2 | Weight | -.324 | .197 | 2.687 | 1 | .101 | .724 | .491 | 1.065 |
|  | Treat | 5.672 | .626 | 82.050 | 1 | .000 | 290.479 | 85.144 | 991.002 |
|  | Phase | -1.056 | .224 | 22.154 | 1 | .000 | .348 | .224 | .540 |
| Step 3 | Treat | 5.600 | .621 | 81.379 | 1 | .000 | 270.338 | 80.082 | 912.595 |
|  | Phase | -.938 | .210 | 20.041 | 1 | .000 | .391 | .259 | .590 |

Method = Backward Stepwise (Wald) in SPSS 13.0

The hazard ratio of solitary to gregarious is around 2.6 times (Exp(B)=1/0.391=2.6)
