# Supplementary material for: Altered Immunity in Crowded Locust Reduced Fungal (Metarhizium anisopliae) Pathogenesis
Source: PLoS Pathog. 2013 Jan 10;9(1):e1003102. doi: 10.1371/journal.ppat.1003102 (PMC3542111; doi:10.1371/journal.ppat.1003102)
Supplement: Table S2 — General features of de novo assembled transcripts by Trinity software. GC: fat body sample of pre-infected gregarious locusts; GI: fat body sample of gregarious locusts infected by fungi M. anisopliae; SC: fat body sample of pre-infected solitary locusts; SI: fat body sample of solitary locusts infected by fungi M. anisopliae; shaded sample was selected as reference transcripts. (DOC) [file ppat.1003102.s013.doc]

| **Samples** | **Reads(M)** | **Assembled**  **transcripts** | **Longest transcripts (kb)** | **N50(bp)** | **BlastX numbers** | **Extracted ORF numbers** |
| --- | --- | --- | --- | --- | --- | --- |
| GC | 142 | 59,062 | 12.680 | 926 | 23,982 | 13,840 |
| GI | 220 | 73,013 | 12.677 | 967 | 28,291 | 16,547 |
| SC | 210 | 76,904 | 16.429 | 1,291 | 28,714 | 17,168 |
| SI | 240 | 68,495 | 21.697 | 962 | 27,881 | 15,737 |

Table S2 General features of *de novo* assembled transcripts by Trinity software
