# Supplementary material for: Altered Immunity in Crowded Locust Reduced Fungal (Metarhizium anisopliae) Pathogenesis
Source: PLoS Pathog. 2013 Jan 10;9(1):e1003102. doi: 10.1371/journal.ppat.1003102 (PMC3542111; doi:10.1371/journal.ppat.1003102)
Supplement: Table S3 — Differentially expressed transcripts were determined by EdgeR software. Different thresholds of significance (adjusted P values) were set for observe differentially expressed transcripts between pre- post-infected samples of the two phases locust. (DOC) [file ppat.1003102.s014.doc]

Table S3 Differential expressed transcripts were determined by EdgeR software

|  | Adjust P value <0.005 | | | Adjust P value <0.01 | | | Adjust P value <0.05 | | |
| --- | --- | --- | --- | --- | --- | --- | --- | --- | --- |
|  | Down | Up | Total | Down | Up | Total | Down | Up | Total |
| **GC vs. SC** | 2,242 | 845 | **3,087** | 2,494 | 977 | **3,471** | 3,670 | 1,532 | **5,202** |
| **GI vs. GC** | 117 | 202 | **319** | 130 | 258 | **388** | 226 | 572 | **798** |
| **SI vs. SC** | 881 | 2,682 | **3,563** | 1,126 | 3,088 | **4,214** | 1,879 | 4,432 | **6,311** |
