# Supplementary material for: Altered Immunity in Crowded Locust Reduced Fungal (Metarhizium anisopliae) Pathogenesis
Source: PLoS Pathog. 2013 Jan 10;9(1):e1003102. doi: 10.1371/journal.ppat.1003102 (PMC3542111; doi:10.1371/journal.ppat.1003102)
Supplement: Table S4 — Differentially expressed transcripts were determined by DEGseq software. Different thresholds of significance (P values) and false discovery rate (FDR, Q-values) were set for observe differentially expressed transcripts between pre- post-infected samples of the two phases locust. (DOC) [file ppat.1003102.s015.doc]

Table S4 Differential expressed transcripts were determined by DEGseq software

|  | Q value <0.005, P<0.001 | | | Q value <0.01, P<0.001 | | | Q value <0.05,P<0.001 | | |
| --- | --- | --- | --- | --- | --- | --- | --- | --- | --- |
|  | Down | Up | Total | Down | Up | Total | Down | Up | Total |
| **GC vs. SC** | 1,134 | 687 | **1,821** | 1,315 | 757 | **2,072** | 1,437 | 807 | **2,244** |
| **GI vs. GC** | 269 | 252 | **521** | 292 | 278 | **570** | 384 | 361 | **745** |
| **SI vs. SC** | 1,302 | 648 | **1,950** | 1,566 | 712 | **2,278** | 1,718 | 735 | **2,453** |
