# Supplementary material for: Altered Immunity in Crowded Locust Reduced Fungal (Metarhizium anisopliae) Pathogenesis
Source: PLoS Pathog. 2013 Jan 10;9(1):e1003102. doi: 10.1371/journal.ppat.1003102 (PMC3542111; doi:10.1371/journal.ppat.1003102)
Supplement: Table S5 — Differentially expressed genes were confirmed by qPCR experiments. Twelve differentially expressed genes determined by RNAseq were confirmed by Q-PCR and showed positive correlation (Pearson r = 0.92). (DOC) [file ppat.1003102.s016.doc]

Table S5 Differentially expressed genes were confirmed by qPCR experiments

| **GENES** | **Fold Change Log_2_(G/S)** | | **DETSs No#** |
| --- | --- | --- | --- |
|  | **RNAseq** | **qPCR** |  |
| GNBP3 | 1.81 | 1.69 | comp614_c0_seq1 |
| PGRP-SA | 1.08 | 1.9 | comp155_c0_seq1 |
| P450 | 1.45 | 2.09 | comp648_c0_seq1 |
| Peroxiredoxin | 6.88 | 5.41 | comp1126_c0_seq1 |
| Serpin | 0.93 | 1.91 | comp47_c0_seq1 |
| Cactus | 2.84 | 4.27 | comp2203_c0_seq2 |
| Hexamerin | -6.16 | -5.07 | comp355_c1_seq1 |
| Glucuronosyltransferases | -2.66 | -4.41 | comp390_c0_seq1 |
| Prolylisomerase | -1.62 | -3.39 | comp289_c0_seq1 |
| NADP-ME | -1.75 | -2.08 | comp461_c0_seq1 |
| GLDH | -5.08 | -2.35 | comp5434_c0_seq1 |
| PSPH | -1.81 | -2.99 | comp824_c0_seq1 |
| *Pearson (r)* | *0.92* | |  |
