# Supplementary material for: Altered Immunity in Crowded Locust Reduced Fungal (Metarhizium anisopliae) Pathogenesis
Source: PLoS Pathog. 2013 Jan 10;9(1):e1003102. doi: 10.1371/journal.ppat.1003102 (PMC3542111; doi:10.1371/journal.ppat.1003102)
Supplement: Table S6 — Primers for Q-PCR and amplification of cDNAs. Reversed (R) and forward (F) primers for Q-PCR and amplification of cDNAs were listed in the table. (DOC) [file ppat.1003102.s017.doc]

**Table S6 Primers for Q-PCR and amplification of cDNAs**

| **Primer Number** | **Sequence (5’🡪3’)** |
| --- | --- |
| **Quantitative real-time PCR** | |
| Q-GNBP1-F | CAGAAGTGGGAACTCACAAAT |
| Q-GNBP1-R | GGTAATCTGGACTTCCTGCTA |
| Q-GNBP3-F | ACCATCAGTGACAAGACCGC |
| Q-GNBP3-R | TTACAGGCAGGCTTTCCATT |
| Q-Toll-F | AATCCTTGGGAATGTGACTGT |
| Q-Toll-R | ATTGCTCTTTGATCTATTACCTT |
| Q-Attacin-F | GTGCTCCTCGTCGTTCTGA |
| Q-Attacin-R | CCCACGCCTTTCTCTCTGT |
| Q-PGRPSA-F | ACCGTGTCGCCCATCT |
| Q-PGRPSA-R | CCCACCAGGAACGAGTAG |
| Q-Actin-F | CGAAACCTTTAATACCCCAG |
| Q-Actin-R | CCATCACCAGAATCCAACAC |
| Q-M3K4-F | CTGTATGTGAGCCCGAAAAGA |
| Q-M3K4-R | GTTGGTCACTGCTGTGCCTAT |
| Q-P450-F  Q-P450-R  Q-Peroxiredoxin-F  Q-Peroxiredoxin-R  Q-Serpin-F  Q-Serpin-R  Q-Cactus-F  Q-Cactus-R  Q- Hexamerin-F  Q- Hexamerin-R  Q- GT-F  Q-GT-R  Q- Prolylisomerase-F  Q- Prolylisomerase-R  Q- NADP-ME-F  Q- NADP-ME-R  Q-GLDH-F  Q-GLDH-R  Q-PSPH-F  Q-PSPH-R | AATCAGGCGTCTACTATTTCC  CAGGCGTTATCATCTTGC  TGAATCGCATAGGTTTGAAGA  CTCATTTCTGGGCTTATTTGT  TGAATCGCATAGGTTTGAAGA  TCGCCTCTGAAGTAAATAGCA  CGTTCGGGTAACACTGCTCT  CCATCGTTTCTTCTGCCACT  CCCTTGTTCAGTATGTCAGC  CCCTTGTTCAGTATGTCAGC  AGCGGCTCTACAACACTTACTT  TTCGTAGGCAGATGGGACA  TGAAACATACTGGACCAGGAAT  CAACAACTGACCCAAATACAAC  TCCCTGGAGTTGCATTAGG  AAGGTGGGTAAAGGCTTCC  CTGCACCTGACATCAACACTG  CTCGTCCATTTATGCCTCCTT  TCTTCATCAGAGAAGCACACC  CAAATCCTGCATAATCACCAT |
| **RACE** | |
| GNBP1-RACE-5: | GCAAGCGTAGATTTCCAAGGCACTGTTG |
| GNBP1-RACE-3: | ACAGTGCCTTGGAAATCTACGCTTGCTC |
| GNBP1-RACE-5-nest: | GTAGATTTCCAAGGCACTGTTG |
| GNBP1-RACE-3-nest: | CTTGGAAATCTACGCTTGCTC |
| GNBP2-RACE-5: | GTTCCCTCCGCCTGACATGGTGATCTCGTG |
| GNBP2-RACE-3: | GCTGACCAGTGTACCAACCCGTCGTACTAC |
| GNBP2-RACE-5-nest: | CCTGACATGGTGATCTCGTG |
| GNBP2-RACE-3-nest: | AGTGTACCAACCCGTCGTACTAC |
| **Recombinant expression in E.coli** | |
| GAPDH1: | CGCGGATCCATGTCGAAGATCGGTATCAAC (BamH I) |
| GAPDH2: | CCGCTCGAGGTCCTTTGACTGCATGTAC (Xho I) |
| GNBP1-P1: | CGCGGATCCGGCCTCAGGATCTCCATACCAG (BamH I) |
| GNBP1-P2: | CCGCTCGAGAGATTTCCAAGGCACTGTTGTTG (Xho I) |
| GNBP1-P3: | CCGCTCGAGCTTAGCACGAATTTCTATAACG (Xho I) |
| GNBP3-NS: | CGCGGATCCGTCCCCGAGCCGCTCATACAG (BamH I) |
| GNBP3-N: | CCGCTCGAGAGCACGGATTTCTATCACACC (Xho I) |
| GNBP3-C: | CGCGGATCCCTCGAAGCAGGTTGCAGACTTG (BamH I) |
| **Recombinant expression in SF9 cell** | |
| GNBP1full P1: | CGCGGATCCATGGAGACCCGCGCGCTGGTTCTGT (BamH I) |
| GNBP1full P2: | CCGCTCGAGCTACATGGCCCATACTTTCACATAA (Xho I) |
| GNBP3full P1: | CGCGGATCCATGCGCGCGTCGCCGCTGCTAGCCT (BamH I) |
| GNBP3full P2: | CCGCTCGAGCTACAGTGCCCACACTTTTACATAG (Xho I) |
| **RNAi** | |
| GFP-RANi-F | CACAAGTTCAGCGTGTCCG |
| GFP-RANi-R | GTTCACCTTGATGCCGTTC |
| GNBP1-RANi-F | GCTGGTTCTGTTGTTGTCGCTG |
| GNBP1-RANi-R | CATTGACCATTGTGTCTGAAGG |
| GNBP3-RANi-F | TCGCCGCTGCTAGCCTTGCTGC |
| GNBP3-RANi-R | CCATTCACAGTGGTGACGGTGC |
| GFP-RANi-F-T7 | TAATACGACTCACTATAGGCACAAGTTCAGCGTGTCCG |
| GFP-RANi-R-T7 | TAATACGACTCACTATAGGGTTCACCTTGATGCCGTTC |
| GNBP1-RANi-F-T7 | TAATACGACTCACTATAGGGCTGGTTCTGTTGTTGTCGCTG |
| GNBP1-RANi-R-T7 | TAATACGACTCACTATAGGCATTGACCATTGTGTCTGAAGG |
| GNBP3-RANi-F-T7 | TAATACGACTCACTATAGGTCGCCGCTGCTAGCCTTGCTGC |
| GNBP3-RANi-R-T7 | TAATACGACTCACTATAGGCCATTCACAGTGGTGACGGTGC |
